# Nicotinamide phosphoribosyltransferase prompts bleomycin-induced pulmonary fibrosis by driving macrophage M2 polarization in mice

**DOI:** 10.7150/thno.94482

**Published:** 2024-04-28

**Authors:** Yaling Chen, Tong Wang, Fuxiang Liang, Jia Han, Zhiling Lou, Yifan Yu, Jinsheng Li, Tianwei Zhan, Yuqing Gu, Lingjun Dong, Bo Jiang, Weiping Zhang, Ming Wu, Yunbi Lu

**Affiliations:** 1 Department of Pharmacology, School of Basic Medical Sciences, Zhejiang University, Hangzhou, Zhejiang Province, China.; 2 Department of Thoracic Surgery, The Second Affiliated Hospital, School of Medicine, Zhejiang University, Hangzhou, Zhejiang Province, China.; 3 Department of Head and Neck Surgery, Zhejiang Provincial People's Hospital, People's Hospital of Hangzhou Medical College, Hangzhou, Zhejiang Province, China.; 4 Department of Pharmacology, School of Pharmacy, Zhejiang University, Hangzhou, Zhejiang Province, China.; 5 Department of Thoracic Surgery, Shaoxing People's Hospital, Shaoxing, Zhejiang Province, China.; 6 Department of Clinical Pharmacology, The Second Affiliated Hospital, School of Medicine, Zhejiang University, Hangzhou, Zhejiang Province, China.

**Keywords:** pulmonary fibrosis, nicotinamide phosphoribosyltransferase, macrophage, M2 polarization, STAT6

## Abstract

**Rationale:** Idiopathic pulmonary fibrosis (IPF) is an irreversible, fatal interstitial lung disease lacking specific therapeutics. Nicotinamide phosphoribosyltransferase (NAMPT), the rate-limiting enzyme of the nicotinamide adenine dinucleotide (NAD) salvage biosynthesis pathway and a cytokine, has been previously reported as a biomarker for lung diseases; however, the role of NAMPT in pulmonary fibrosis has not been elucidated.

**Methods:** We identified the NAMPT level changes in pulmonary fibrosis by analyzing public RNA-Seq databases, verified in collected clinical samples and mice pulmonary fibrosis model by Western blotting, qRT-PCR, ELISA and Immunohistochemical staining. We investigated the role and mechanism of NAMPT in lung fibrosis by using pharmacological inhibition on NAMPT and *Nampt* transgenic mice. *In vivo* macrophage depletion by clodronate liposomes and reinfusion of IL-4-induced M2 bone marrow-derived macrophages (BMDMs) from wild-type mice, combined with *in vitro* cell experiments, were performed to further validate the mechanism underlying NAMPT involving lung fibrosis.

**Results:** We found that NAMPT increased in the lungs of patients with IPF and mice with bleomycin (BLM)-induced pulmonary fibrosis. NAMPT inhibitor FK866 alleviated BLM-induced pulmonary fibrosis in mice and significantly reduced NAMPT levels in bronchoalveolar lavage fluid (BALF). The lung single-cell RNA sequencing showed that NAMPT expression in monocytes/macrophages of IPF patients was much higher than in other lung cells. Knocking out NAMPT in mouse monocytes/macrophages (*Nampt^fl/fl^;Cx3cr1^CreER^*) significantly alleviated BLM-induced pulmonary fibrosis in mice, decreased NAMPT levels in BALF, reduced the infiltration of M2 macrophages in the lungs and improved mice survival. Depleting monocytes/macrophages in *Nampt^fl/fl^;Cx3cr1^CreER^* mice by clodronate liposomes and subsequent pulmonary reinfusion of IL-4-induced M2 BMDMs from wild-type mice, reversed the protective effect of monocyte/macrophage NAMPT-deletion on lung fibrosis. *In vitro* experiments confirmed that the mechanism of NAMPT engaged in pulmonary fibrosis is related to the released NAMPT by macrophages promoting M2 polarization in a non-enzyme-dependent manner by activating the STAT6 signal pathway.

**Conclusions:** NAMPT prompts bleomycin-induced pulmonary fibrosis by driving macrophage M2 polarization in mice. Targeting the NAMPT of monocytes/macrophages is a promising strategy for treating pulmonary fibrosis.

## Introduction

Idiopathic pulmonary fibrosis (IPF) is the most common interstitial lung disease characterized by irreversible impairment of lung function and poor prognosis 1, with a median survival of 2-4 years after diagnosis 2. It is believed that multiple genetic- and non-genetic risk interactions prompt inflammation, scarring, fibrosis, and matrix deposition in the IPF lungs chronically and irreversibly, ultimately leading to respiratory failure and death 3, 4. Up to now, the specific therapeutic drugs that reverse IPF or halt the progression of IPF are still unavailable due to the incomplete understanding of the pathogenesis of IPF 5.

Pulmonary fibrosis begins with the dysregulation of lung tissue repair triggered by repeated injury on alveoli epithelial cells 6. As one of the foremost innate and acquired immunity controllers, macrophages orchestrate tissue homeostasis and plasticity following epithelial cell damage and activate throughout the pathological process of pulmonary fibrosis 7. Macrophages activate to remove pathogens and cellular debris, and produce reactive oxygen species and inflammatory mediators to recruit more immune cells 8. Massive circulating monocytes are recruited into the lung and differentiate into macrophages 9. Macrophages also recruit fibroblasts to form fibrotic microenvironment 10 and produce profibrotic factors to promote fibrosis 11.

According to the responses to different stimuli *in vitro*, the highly plastic macrophages are classified into a classically activated phenotype (M1) or an alternatively activated phenotype (M2). Although this dichotomy is now highly debated in many inflammatory diseases, the M1/M2 functional classification still contributes to understanding the role of macrophages in IPF. Specifically, M1 macrophages produce large amounts of pro-inflammatory cytokines and chemokines (including TNF-α and IL-6), as well as pathogen-associated molecular patterns (PAMPs), which drive inflammation and tissue damage. If the factors causing tissue damage are not addressed, tissue inflammation induces macrophage polarization towards the M2 type, which can produce TGF-β to promote tissue remodeling and extracellular matrix deposition, driving the progression of pulmonary fibrosis. IPF patients are predominantly infiltrated by M2 macrophages during disease progression 12, 13. Thus, intervention of macrophage M2 polarization could be a potential strategy for IPF 14, 15. However, the regulation mechanism of macrophage polarization in pulmonary fibrosis is unclear.

Nicotinamide phosphoribosyltransferase (NAMPT), also known as visfatin or pre-B-cell clonogenic enhancer factor (PBEF), is highly conserved in living organisms 16. NAMPT has intracellular and extracellular forms in mammals. The intracellular NAMPT (iNAMPT) is the bottleneck enzyme of the nicotinamide adenine dinucleotide (NAD) salvage biosynthesis pathway 17. Extracellular NAMPT (eNAMPT) functions as an inflammatory factor 18. NAMPT is involved in the pathogenesis of many inflammatory diseases, including atherosclerosis, intestinal inflammation, and cerebral ischemia, by driving macrophage polarization 19-21. During acute lung injury 22 and pulmonary arterial hypertension 23, NAMPT increases, and eNAMPT neutralization mitigates lung injuries. However, the role of NAMPT in pulmonary fibrosis has not been elucidated.

In this study, we found that NAMPT was upregulated and released from macrophages during pulmonary fibrosis. NAMPT promoted lung fibrosis by driving macrophage M2 polarization in an enzymatic-independent, STAT6-dependent manner. Targeting the NAMPT of monocytes/macrophages is a new potential strategy for treating pulmonary fibrosis.

## Methods

### Human lung tissue samples

Human pulmonary fibrosis tissue samples (N = 7) were collected from patients with confirmed pulmonary fibrosis who underwent lung transplantation at the Second Affiliated Hospital of Zhejiang University School of Medicine in 2021 and 2022. Control lung tissue samples (N = 6) were obtained from donors for single lung transplantation at the Second Affiliated Hospital of Zhejiang University School of Medicine in 2021 and 2022. The Ethics Committee of the Second Affiliated Hospital of Zhejiang University School of Medicine approved all experimental protocols (IRB-2021-11). Written informed consent was received from participants prior to inclusion in the study.

### Animals

All experiments were performed in male mice because male animals exhibited less variability in phenotype. Wild-type C57BL/6J mice (male, 8-12 weeks, 23-26 g) were purchased from the Experimental Animal Center, Zhejiang Academy of Medical Sciences (Hangzhou, China). *Nampt^flox/flox^* mice were custom-made by Nanjing Biomedical Research Institute of Nanjing University (Nanjing, China) using the Cre-LoxP system as previously described 24. NAMPT conditional knockout (cKO) mice (genotype: *Nampt^flox/flox^;Cx3cr1^CreER^*) with tamoxifen-inducible knockout of NAMPT in monocytes/macrophages were obtained by crossing the *Cx3cr1^CreER^* mice (gifted by Professor Shumin Duan, Zhejiang University School of Medicine) and *Nampt^flox/flox^* mice. *Nampt^flox/flox^* mice served as age- and sex-matched wild-type (WT) controls for NAMPT cKO mice and received the same tamoxifen treatments.

The mice were kept in the Laboratory Animal Center of Zhejiang University. The mice had free access to water and food in a specific pathogen-free (SPF) environment with room temperature at 23-25 °C and relative humidity of 50% on a 12 h light/dark cycle. The experimental protocols were approved by Laboratory Animal Welfare and Ethics Committee of Zhejiang University (ZJU20210287). All mice were handled following the Guide for the Care and Use of the Laboratory Animals of the National Institutes of Health.

### Bleomycin-induced lung fibrosis model

Mice were given 3 mg/kg bleomycin (BLM, Cat#T6116, Topscience, Shanghai, China) in 50 μL sterile saline intratracheally to induce lung fibrosis. The sham group received an equal volume of sterile saline via intratracheal injection. Mice were randomly grouped. The day of modeling was defined as Day 0. All mice were sacrificed 14 days after modeling (Day 14).

### Administration of FK866

FK866 (Cat#658084-64-1, Selleck Chemicals, Shanghai, China), the specific inhibitor of NAMPT, was dissolved in DMSO and stocked at 40 mg/mL. The stock solution of FK866 was diluted in sterile saline and intraperitoneally injected once per day for 16 days, from Day -1 to Day 14. The second dose was administered 30 min after bleomycin injection. The same volume of vehicle was intraperitoneally administered to the control group. For dose-response studies, FK866 was administered at 1 mg/kg, 3 mg/kg or 10 mg/kg with a final concentration of DMSO of less than 1%.

### Administration of Tamoxifen

Tamoxifen (TAM, Cat#MB1233, Meilun Bio., Dalian, China) was dissolved in sterile saline and sonicated for 30 min to prepare a suspension at a concentration of 20 mg/mL. The first two tamoxifen doses (10 mg/dose) were administrated by gavage at 16 days and 14 days before modeling, respectively. The third dose was administered on the day before modeling.

### Bone Marrow Derived Macrophage (BMDM)

Mice were sacrificed and immersed in 75% alcohol for 5 min. We separated the tibia and femur of the limbs under sterile conditions, cut both ends of the bone, and washed the bone cavity repeatedly with Dulbecco's Modified Eagle Medium (DMEM, Cat#C11995500BT, Gibco, USA) until it was transparent. We filtered the cells with a 70-µm cell strainer (Cat#BS-70-XBS, Biosharp, Hefei, China). Red blood cells were lysed and removed. After centrifugation, the cells were collected and cultured in DMEM containing 40 μg/mL recombinant mouse M-CSF (Cat#315-02, PeproTech, Suzhou, China). We replaced half of the complete culture medium three days later with fresh DMEM containing M-CSF. We completely replaced the culture medium on Day 5 and continued culturing for 1-2 days to obtain primary bone marrow macrophages for further study.

### Macrophage depletion

Clodronate liposomes (Cat#F70101C-NC, FormuMax, California, US) 200 μL were injected into the tail vein one day before BLM induction (control liposomes were used as a control). Macrophage depletion was induced utilizing the phagocytosis of macrophages. The lungs of mice were isolated for Western blotting and pathological analysis 14 days after BLM induction.

### Macrophage adoptive transfer

BMDMs from WT mice were stimulated with 20 ng/mL recombinant mouse IL-4 (Cat#214-14-20, PeproTech, Suzhou, China) for 12 h to induce M2 polarization. Lung fibrosis was induced using BLM in WT and NAMPT cKO mice after macrophage depletion. On Day 7 of BLM induction, M2-polarized BMDMs (1×10^6^ cells/mouse, 50 μL) were injected intratracheally. Mice were sacrificed after one week for lung fibrosis analysis.

### HE staining and Masson's trichrome staining

The left lung of mice was embedded in paraffin after fixing for 24 h in 10% formaldehyde. The paraffin-embedded lung tissue was cut into 4-μm sections. The slices were treated with HE or Masson's trichrome staining. The inflammatory cell infiltration and other inflammatory changes were observed after HE staining. As previously described 25, the peribronchial regions in four different fields of one slide were scored at 200× magnification (0, normal; 1, < 3 cells diameter thick; 2, 3-10 cells thick; 3, > 10 cells thick). Advanced Ashcroft scoring system 26 was used to evaluate the degree of pulmonary fibrosis based on Masson's trichome staining. Grade 0 indicated lung tissue without fibrosis, and grades 1-8 illustrated fibrosis levels from minor to severe. The scoring was performed by a technician who did not know the experimental design.

### Immunohistochemistry

Paraffin-embedded lung sections were immersed in xylene twice for 15 min to remove wax. The sections were then hydrated twice by sequentially bathing them in 100%, 95% and 70% ethanol and ddH_2_O for 5 min. After soaking in 3% H_2_O_2_ for 15 min, the slides were subjected to antigen retrieval (pH 6 sodium citrate buffer or pH 9 EDTA buffer) and then incubated with rabbit anti-NAMPT monoclonal antibody (Cat#A300-372A, BETHYL, Texas, USA; 1:500) overnight at 4 °C. The DAB Substrate System (Cat#P0612, Beyotime Bio, Shanghai, China) was used according to the manual to reveal the immunohistochemical staining the next day. And cell nuclei were counterstained with hematoxylin. Images were captured under a microscope (BX51, Olympus, Japan).

### Immunofluorescence

After routine dewaxing, hydration, and antigen retrieval, the lung slices were incubated overnight at 4 °C with the following primary antibodies: rabbit anti-NAMPT (Cat#A300-372A, BETHYL, Texas, USA; 1:500); rat anti-F4/80 (Cat#ab6640, Abcam, Cambridge, UK; 1:100); mouse anti-CD68 (Cat#ab955, Abcam, Cambridge, UK; 1:200); rabbit anti-Cre (Cat#15036, Cell Signaling Technology, Massachusetts, USA; 1:200); mouse anti-Cre (Cat#MAB3120, Sigma-Aldrich, Missouri, USA; 1:200); rabbit anti-CD86 (Cat#19589, Cell Signaling Technology, Massachusetts, USA; 1:100); rabbit anti-CD206 (Cat#ab64693, Abcam, Cambridge, UK; 1:250).

For RAW264.7 cells, immunofluorescence staining was used to assess the nuclear translocation of STAT6. Briefly, the cells attached to glass were sequentially fixed with 4% PFA for 15 min, washed three times with phosphate buffer saline (PBS) for 3 min each time, permeabilized with 0.1% Triton X-100 for 20 min, and blocked with 5% normal donkey serum (NDS) for 30 min. Then, the cells were incubated with rabbit anti-STAT6 antibody (Cat#ab32520, Abcam, Cambridge, UK; 1:100) at 4 °C overnight.

The next day, the slices and the cells were washed with PBS and then incubated with secondary antibodies: Cy3-conjugated donkey anti-mouse antibody (Cat#AP129); Cy3-conjugated donkey anti-rabbit antibody (Cat#AP182C); Cy3-conjugated donkey anti-rat antibody (Cat#AP189C); FITC-conjugated goat anti-mouse antibody (Cat#AP124F); FITC-conjugated goat anti-rabbit antibody (Cat#AP132F); FITC-conjugated goat anti-rat antibody (Cat#AP183F), which were all purchased from Millipore, Massachusetts, USA, and used at a dilution of 1:200. Nuclei were stained with DAPI (Cat#P36935, Thermo Fisher Scientific, Massachusetts, USA). The images were observed and graphed under confocal fluorescence microscope (FV100, Olympus, Japan).

### Cell culture and treatment

RAW264.7 cells (ATCC), a murine leukemic monocyte/macrophage cell line, were cultured in high glucose DMEM (Cat#C11995500BT, Gibco, USA), supplemented with 10% heat-inactivated Fetal Bovine Serum (Cat#10091-148, Gibco, USA) and 1% penicillin-streptomycin (Cat#P1400, Solarbio, Beijing, China) at 37 °C in a humidified 5% CO_2_ atmosphere.

Cells were seeded at a density of 4 × 10^5^ cells/well in six-well plates. Adherent cells were pretreated with 1 nM FK866 (Cat#658084-64-1, Selleck Chemicals, Shanghai, China), 100 μΜ β-NMN (Cat#HY-F0004, MCE, USA) or both for 30 min, or NAMPT antibody for 30 min, and then stimulated with 20 ng/mL recombinant mouse TGF-β1 (Cat#P00199, Solarbio, Beijing, China) for 24 h. Cells or culture supernatants were then collected for subsequent detection.

Cells were seeded at a density of 4 × 10^5^ cells/well in six-well plates or 2 × 10^4^ cells/well in 24-well plates. Adherent cells were pre-treated with 10 nM AS1517499 (Cat#S8685, Selleck, USA) for 1 h and then stimulated with 300 nM recombinant human NAMPT protein 27. After incubation for 24 h, the cells were collected for subsequent detection.

### Cell viability

Cell Counting Kit-8 (CCK-8, Cat#C0038, Beyotime Bio, Shanghai, China) was used to evaluate the effects of FK866, β-NMN, and AS1517499 on the viability of RAW264.7 cells according to the manufacturer's instructions. Briefly, cells were seeded in 96-well plates at a density of 4×10^3^ cells per well. After 24 h of growth, the cells were treated with a variety of concentrations of drugs for the indicated time. Following incubation of the cells with CCK-8 solution for 2 h, the optical density was measured at 450 nm using a microplate reader (Elx800, Bio-TEK, USA). Cell viability was expressed as a percentage of the control.

### siRNA transfection

RAW264.7 cells (5 × 10^5^ cells/well) were cultured in six-well plates and then transfected at 50-70% confluence with 150 pmol NAMPT siRNA or negative control siRNA (NC siRNA) using 8 μL Lipofectamine 2000 (Cat#11668019; Invitrogen, USA) according to the manufacturer's protocols. After transfection for 24 h, cells were treated with 20 ng/mL TGF-β1 for 24 h. Then, the cells or the culture supernatants were collected for the subsequent detections. NAMPT siRNA and NC siRNA were designed at Shanghai GenePharma Co., Ltd (China). The sequences of siRNA are provided as follows.

NAMPT siRNA (5' to 3')

Sense: GCUGCCACCUUAUCUUAGATT

Antisense: UCUAAGAUAAGGUGGCAGCTT

NC siRNA (5' to 3')

Sense: UUCUCCGAACGUGUCACGUTT

Antisense: ACGUGACACGUUCGGAGAATT

### Total NAD quantification

According to the manufacturer's instructions, total NAD levels in mouse tissues, bone marrow cells, and RAW264.7 cells were measured using the NAD^+^/NADH assay kit (Cat#ab65348, Abcam, Cambridge, UK). The protein concentration of samples was determined by BCA Protein Assay Kit (Cat#P0009, Beyotime Bio, Shanghai, China).

### Quantitative Real-Time Polymerase Chain Reaction (RT-PCR) analysis

At the end of treatment, total RNA was extracted from cultured cells or lung tissues using RNAiso PLUS reagent (Takara Biotechnology, Dalian, China). And cDNA was reverse transcribed from RNA using the PrimeScriptTM^RT^ reagent Kit (Takara Biotechnology, Dalian, China). The sequences of primer (Sangon Biotech, Shanghai, China) for qRT-PCR are provided in Table 1. Quantitative PCR was conducted in triplicate for each gene of interest using the SYBR Green kit (Takara Biotechnology, Dalian, China) on LightCycler 480 real-time PCR detection system (Roche, Switzerland). The relative quantification of mRNA expression was calculated using the 2^-∆∆Ct^ method after normalization to *Actb*.

### Enzyme linked immunosorbent assay (ELISA)

Bronchoalveolar lavage was performed with 1.2 mL cold PBS. The concentrations of cytokine in mouse bronchoalveolar lavage fluid (BALF), serum, and the culture supernatant of RAW264.7 cells were measured by ELISA. The TGF-β1 (Cat#EK0515), IL-6 (Cat#EK0411), and TNF-α (Cat#EK0527) ELISA kits were purchased from Wuhan Boster Biological Technology, Ltd., China. The eNAMPT ELISA kit (Cat#MM-44780M1) was purchased from Jiangsu Meimian Biological Technology, Ltd., China.

### Flow Cytometry

Lung tissues were minced with scissors and digested in RPMI-1640 medium (Cat#C11875500BT, Gibco, USA) containing 1 mg/mL collagenase I (Cat#C0130, Sigma-Aldrich, Missouri, USA) and 100 μg/mL DNase (Cat#9003-98-9, Sigma-Aldrich, Missouri, USA) on a shaker at 37 °C for 40 min. The digested lung tissues were filtered through a 70-µm cell strainer. Red blood cells were removed with RBC lysis buffer (Cat#C3702, Beyotime Bio, Shanghai, China). The single-cell suspensions were blocked with anti-mouse CD16/32 (Cat#156603, Biolegend, California, USA) for 15 min and stained with zombie dyes (Cat#423101, Biolegend, California, USA) to distinguish dead from living cells. The cells were washed with cell staining buffer (Cat#420201, Biolegend, California, USA) and incubated with the following antibodies for 30 min at 4 °C: Alexa Fluor 700 rat anti-mouse CD45 (Cat#560510), BV421 rat anti-mouse F4/80 (Cat#565411), BV650 rat anti-mouse CD11b (Cat#563402), PE-Cy7 rat anti-mouse CD86 (Cat#560582), which were all purchased from BD Pharmingen, Ltd., San Diego, USA. After permeabilization and fixation with Cytofix/Cytoper fixation/permeabilization kit (Cat#554714, BD Pharmingen, Ltd., San Diego, USA), cells were incubated with PE rat anti-mouse CD206 (Cat#141706, Biolegend, California, USA) for 30 min. Cells were resuspended in PBS and analyzed on a CytoFLEX flow cytometer (Beckman, USA). To detect Cre expression in monocytes/macrophages, peripheral blood cells were stained with Alexa Fluor 700 rat anti-mouse CD45 (Cat#560510, BD Pharmingen, Ltd., San Diego, USA) and APC anti-mouse Ly6C (Cat#517-5932-82, eBioscience, California, USA**)** after red blood cell lysis and Fc blocking. Data were analyzed with FlowJo (BD Biosciences, USA).

### Western blotting analysis

Total protein was extracted using NP40 (Cat#P0013F) or RIPA lysis buffer (Cat#P0013B) containing protease inhibitor (Cat#P1005-1), phosphatase inhibitor (Cat#P1045-2) and PMSF (Cat#ST506), which were all purchased from Beyotime Bio, Shanghai, China. Lysates were collected and centrifuged at 12,000 rpm for 30 min. Protein concentration was determined using the BCA protein assay kit (Cat#P0009, Beyotime Bio, Shanghai, China). After mixing with loading buffer and heating at 100 °C for 5 min, 30-50 μg protein was loaded into the well of 10% SDS-PAGE gel. The gel was run at 90 V for 2 h, and then the protein was transferred to the nitrocellulose membrane (Cat#PN66485, Pall Corporation, USA). The membrane was blocked with 5% non-fat milk buffer (Cat# A600669, Sangon Biotech, Shanghai, China) for 2 h at room temperature and then incubated overnight at 4 °C with the following primary antibodies at the manufacturer's recommended dilutions: Mouse anti-GAPDH (Cat#60004-1-Ig, Proteintech, Wuhan, China; 1:5000), Mouse anti-β-actin (Cat#66009-1-Ig, Proteintech, Wuhan, China; 1:5000), Rabbit anti-NAMPT (Cat#A300-372M, BETHYL, Texas, USA; 1:10,000), Rabbit anti-Fibronectin (Cat#15613-1-AP, Proteintech, Wuhan, China; 1:5000), Rabbit anti-STAT6 (Cat#ab32520, Abcam, Cambridge, UK; 1:2000), Rabbit anti-pSTAT6 (Tyr641) (Cat#56554, Cell Signaling Technology, Massachusetts, USA; 1:1000). Membranes were probed with HRP-conjugated goat anti-mouse IgG (Cat#7076S, Cell Signaling Technology, Massachusetts, USA; 1:10,000) or HRP-conjugated goat anti-rabbit IgG (Cat#111-035-003, Jackson ImmunoResearch, Pennsylvania, USA; 1:10,000), and visualized by enhanced chemiluminescence on ChemiDoc Touch Imaging System (Bio-Rad, Canada). The intensity of the protein bands was analyzed using Image Lab software (Bio-Rad, Canada) and then normalized to that of the GAPDH or β-actin band.

### RNA-seq assay and bioinformatics analysis

Total RNA was isolated from lung tissues procured from euthanized mice (*Nampt^fl/fl^* and *Nampt^fl/fl^;Cx3cr1^CreER^*, n = 3 per group) 14 days after bleomycin-induced pulmonary fibrosis modeling. Poly (A) RNA was purified from 1 μg total RNA using Dynabeads Oligo (dT)25-61005 (Thermo Fisher, CA, USA) and then fragmented into small pieces using Magnesium RNA Fragmentation Module (Cat#e6150, NEB, USA) under 94 ℃ for 5-7 min. The cleaved RNA fragments were reverse transcribed to generate cDNA and then used to synthesize U-labeled second-stranded DNAs with E. coli DNA polymerase I (Cat#m0209, NEB, USA), RNase H (Cat#m0297, NEB, USA) and dUTP Solution (Cat#R0133, Thermo Fisher, USA). An A-base was then added to the blunt ends of each strand, preparing them for ligation to the indexed adapters. Each adapter contained a T-base overhang for ligating the adapter to the A-tailed fragmented DNA. Single- or dual-index adapters were ligated to the fragments, and size selection was performed with AMPureXP beads. After the heat-labile UDG enzyme (Cat#m0280, NEB, USA) treatment of the U-labeled second-stranded DNAs, the ligated products were amplified with PCR under the following conditions: initial denaturation at 95 ℃ for 3 min; 8 cycles of denaturation at 98 ℃ for 15 sec, annealing at 60 ℃ for 15 sec, and extension at 72 ℃ for 30 sec; and then final extension at 72 ℃ for 5 min. The average insert size for the final cDNA library was 300 ± 50 bp. At last, we performed the 2 × 150 bp paired-end sequencing (PE150) on an Illumina Novaseq™ 6000 (LC-Bio Technology CO., Ltd., Hangzhou, China) following the vendor's recommended protocol.

We used fastp software to remove the reads that contained adaptor contamination, low-quality bases and undetermined bases with default parameters. The sequence quality was also verified using fastp. We used HISAT2 to map reads to the reference genome of *Mus musculus* GRCm38. The mapped reads of each sample were assembled using StringTie with default parameters. Then, all transcriptomes from all samples were merged to reconstruct a comprehensive transcriptome using gffcompare. After the final transcriptome was generated, StringTie was used to estimate the expression levels of all transcripts and perform expression level for mRNAs by calculating FPKM. The differentially expressed mRNAs were selected with fold change > 2 or fold change < 0.5 and with parametric F-test comparing nested linear models (p value < 0.05) by R package edgeR.

### Analysis of public bulk RNA-seq data

The raw transcriptomic data used in this study were retrieved from the Gene Expression Omnibus. GSE72073 consists of the data from the lung tissues of 3 healthy donors and 5 IPF patients. GSE53845 consists of the data from 8 healthy samples and 40 IPF samples. In GSE110147, only healthy donor lung samples (N = 22) and samples with IPF from the interstitial lung disease population (N = 22) were included for analysis. The GEO query package was used to download the series matrix files of the databases above in R (version 4.2.2).

We screened differentially expressed genes (DEGs) between IPF patients (IPF) and healthy donors (HD) using the R package of the Microarray Data Linear Model (limma, version 3.54.2) 28. The significant DEGs were identified according to the thresholds of adjusted P < 0.001 and fold change (FC) > 2 or < 0.5. The common DEGs in the datasets were visualized by the R package ggplot2 (version 3.4.1).

### Analysis of public single-cell RNA-seq data

The single-cell dataset (GSE135893) was retrieved from Gene Expression Omnibus. GSE135893 consists of lung tissues from 10 healthy donors (HD) and 12 IPF patients (IPF). Cluster information can be obtained from the database. The analysis was performed using the R package Seurat (version 4.3.0) 29-32. The heatmap was visualized by the R package Circlize (version 0.4.15) 33 and Complex Heatmap (version 2.14.0) 34, 35.

We isolated macrophages and monocytes from the IPF group in the database for analysis. According to the expression level of NAMPT, these cells were divided into a high expression group (> the upper quartile of NAMPT expression) and a low expression group (< the lower quartile of NAMPT expression). To identify DEGs between cell types, we used a negative binomial model implemented in the Seurat FindMarkers function. Gene Ontology biological process enrichment was performed using the R package cluster Profiler (version 4.7.1.003) 36.

### Statistical analysis

All data are presented as mean ± SD, and were analyzed and plotted using GraphPad Prism 6.0 software (GraphPad Software, CA, USA). The “n” represents the number of mice used in each group, or the number of replicates for *in vitro* cell experiments. Grubbs' test was used to identify outliers. One-way ANOVA was used to analyze the differences among groups. Unpaired *t*-test was applied to analyze the differences between the two groups. Weight curve and survival curve were analyzed using two-way ANOVA. P < 0.05 was considered as statistical significance. The detailed statistical analysis description is given in each figure legend.

## Results

### NAMPT is upregulated in human and mouse pulmonary fibrotic lungs

**Figure 1:**
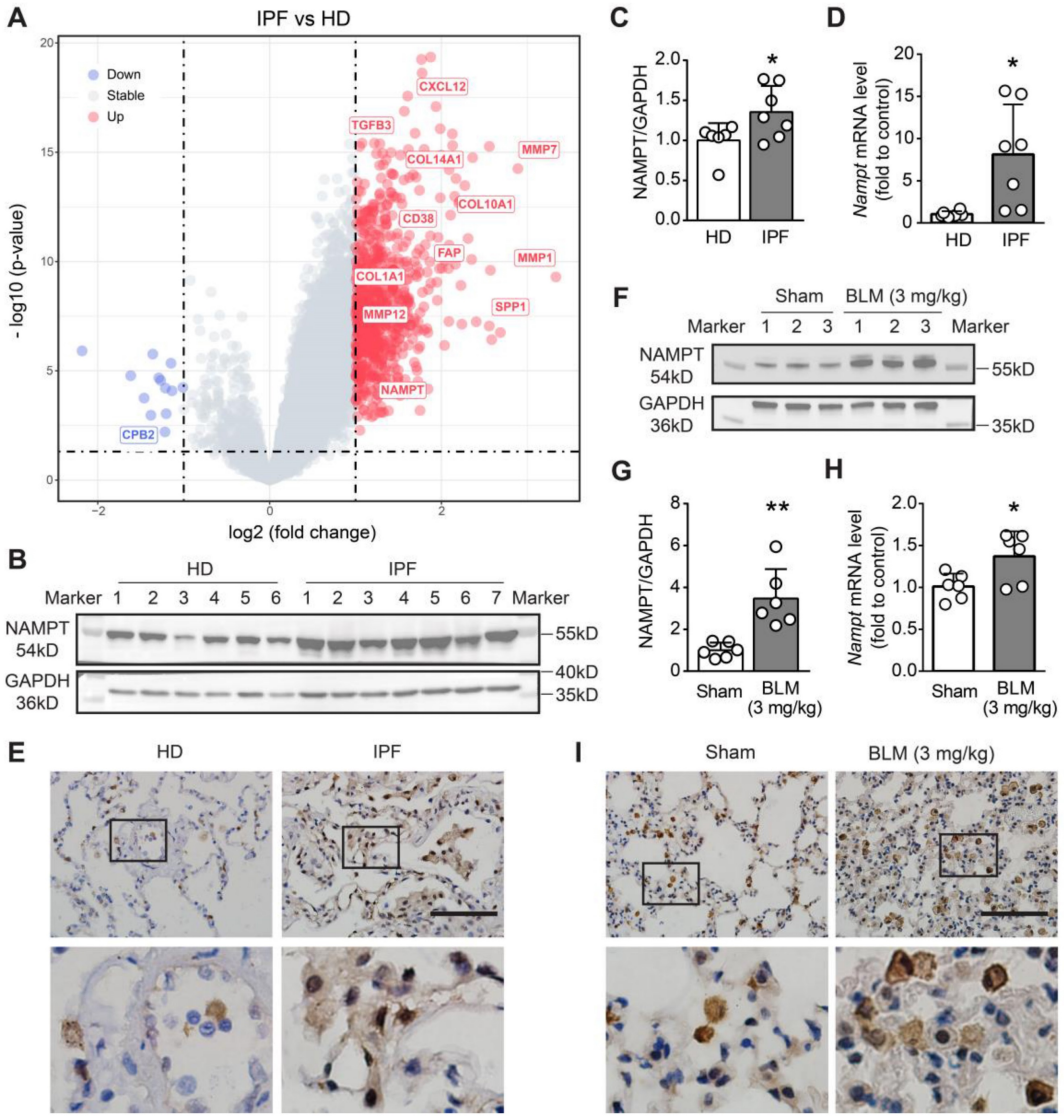
**NAMPT is upregulated in human and mouse fibrotic lungs. (A)** Volcanic map of differentially expressed genes (DEGs) in the lung of idiopathic pulmonary fibrosis (IPF) patients compared to that of healthy donors (HD). Transcriptomic data sources: GSE110147, GSE72073, GSE53845. **(B-C)** Western blotting analysis (B) and quantification (C) for the expression of NAMPT in the lungs of HD and IPF patients (n = 6-7). **(D)** Relative mRNA expression of NAMPT in the lungs of HD and IPF patients. (n = 6-7). **(E)** Immunohistochemical staining of NAMPT in the human lung sections. Bar = 100 μm (n = 3). **(F-I)** The mouse lung tissue was sampled at 14 days after bleomycin (BLM, 3 mg/kg) intratracheal administration. Western blotting analysis **(F)** and quantification **(G)** for the expression of NAMPT (n = 6). **(H)** Relative mRNA expression of NAMPT of the mouse lungs (n = 6). **(I)** Immunohistochemical staining of NAMPT. Bar = 100 μm (n = 3). Mean ± SD, *P < 0.05, **P < 0.01, unpaired* t*-test.

To determine the NAMPT level changes in patients with pulmonary fibrosis, we searched and retrieved three sets of transcriptomic data (GSE72073, GSE53845, GSE110147) from the GEO database and screened the differentially expressed genes (DEGs) in the lung between IPF patients and healthy donors (HD). After data background correction, data normalization and batch effect exclusion were performed. The R package “limma” was used to determine the DEGs. Based on the threshold of adjusted P < 0.001 and fold change (FC) > 2 or < 0.5, four hundred and thirty DEGs were identified, including several cell migration- and fibrosis-associated genes, such as matrix metalloprotein (MMP1, MMP7, MMP12), SPP1, FAP, CXCL12, collagen family (COL10A1, COL14A1, COL1A1). Among these DEGs, we found that NAMPT was also one of the genes whose transcript level was significantly increased in the lungs of IPF patients (Figure 1A).

Then, we determined NAMPT expression levels in the lung tissues of healthy donors (HD) and IPF patients using Western blotting and qRT-PCR. The results showed that NAMPT protein expression and mRNA levels were markedly higher in IPF patients than in HD (Figure 1B-D). Immunohistochemical staining showed that NAMPT-positive cells significantly increased (Figure 1E), indicating elevated NAMPT expression in the IPF lung. We further induced lung fibrosis in mice by intratracheal injection of bleomycin (BLM, 3 mg/kg), according to the previous report 37. Similarly, we observed an upregulation of NAMPT in fibrotic mouse lungs (Figure 1F-I). Here, we collected mouse lung samples after pulmonary artery perfusion with saline to avoid interference from blood cells and then extracted tissue proteins for blotting assay and qRT-PCR. Furthermore, we detected the NAMPT expression during pulmonary fibrosis at 7 days, 14 days, 21 days, and 28 days after BLM administration in mice, and we found that NAMPT expression peaked at 14-21 days (Figure S1) when the lung injury and fibrosis became severe. Therefore, in the subsequent experiments, the mice were treated with bleomycin (3 mg/kg) and sacrificed for sample collection on day 14.

### NAMPT inhibitor FK866 ameliorates bleomycin-induced pulmonary fibrosis in mice

**Figure 2:**
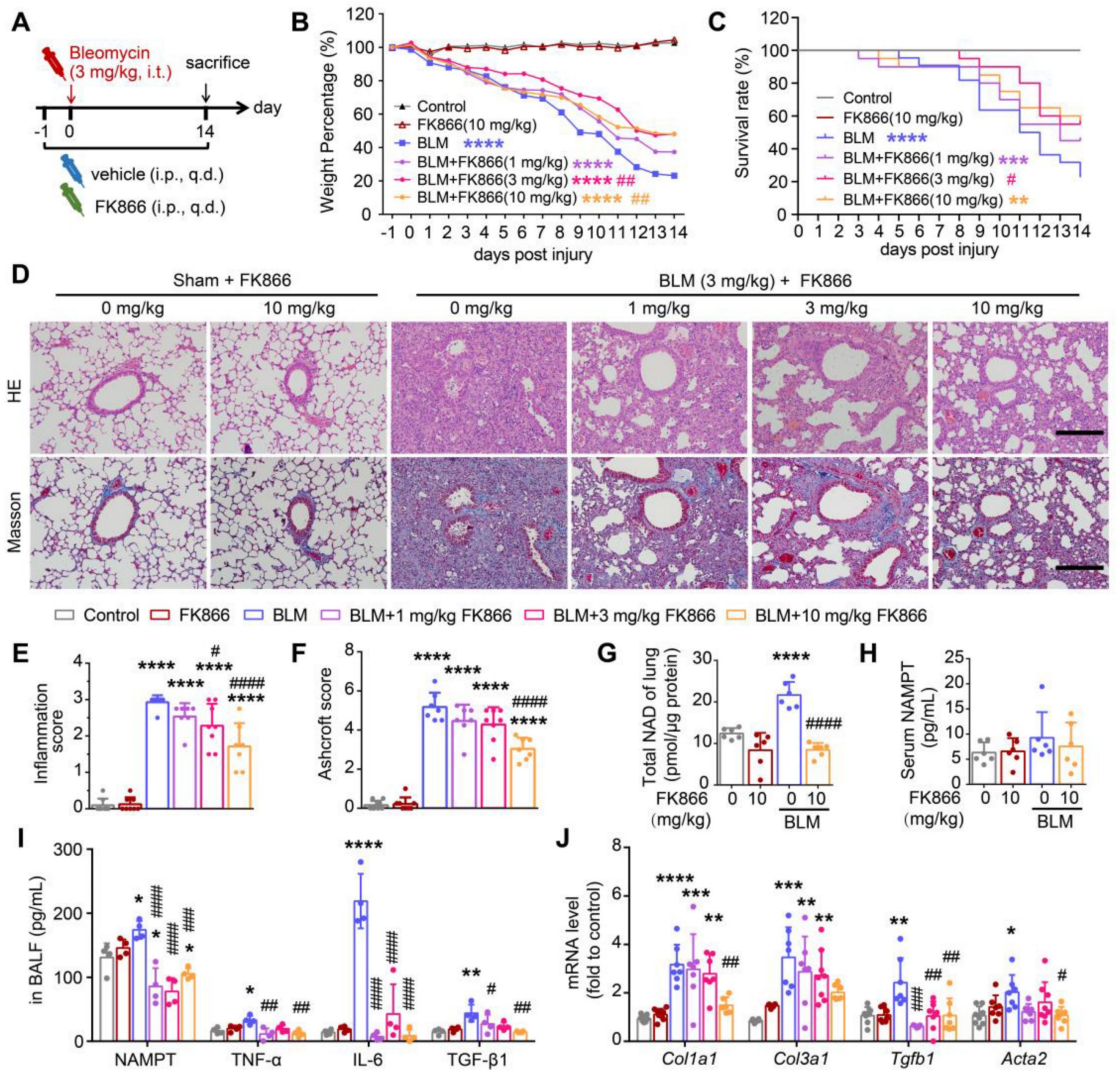
** NAMPT inhibitor FK866 mitigates bleomycin-induced lung fibrotic injury in mice. (A)** Schematic draw of the experimental design. Bleomycin (BLM, 3 mg/kg) and FK866 (1, 3, 10 mg/kg) were administrated via intratracheal and intraperitoneal injection, respectively. **(B)** The mouse body weight was recorded at the indicated time and shown as percentage of the baseline body weight. **(C)** Kaplan-Meier curves for survival rate of the mice. Control group, n = 12; FK866 alone group, n = 12; BLM administrated group, n = 20-22. **(D)** Representative graphs of HE and Masson's trichrome staining of mouse lungs. Bar = 200 μm. **(E, F)** The development of lung lesions revealed by HE and Masson's trichrome staining was scored according to the double-blind principle (n = 7-8). **(G-H)** The NAD levels in the lung tissue (G) and the extracellular NAMPT levels in the serum (H) were determined using ELISA (n = 6). **(I)** ELISA analysis of extracellular NAMPT, TNF-α, IL-6, and TGF-β1 in BALF (n = 4). **(J)** Relative mRNA expression of the collagen factors (*Col1a1, Col3a1, Tgfb1* and *Acta2*) (n = 7-8). Mean ± SD, *P < 0.05, **P < 0.01, ***P < 0.001, ****P < 0.0001, compared with Control. *^#^P* < 0.05, *^##^P* < 0.01, *^###^P* < 0.001, *^####^P* < 0.0001, compared with BLM, one-way ANOVA.

To preliminarily elucidate the role of NAMPT in lung fibrosis, we used FK866, an enzymatic inhibitor of NAMPT 38. Mice were injected intraperitoneally with various doses of FK866 (1, 3, 10 mg/kg) or an equal volume of the vehicle once a day, starting the day before modeling with bleomycin (3 mg/kg), and the mice were sacrificed for sample collection on day 14 after modeling (Figure 2A).

Through the tracking observation of mice in each group, we found that the mice in the control group (Control) and FK866 alone treatment group (FK866) had moist hair and no apparent differences in body weight at each indicated time point. The bleomycin-treated mice (BLM) had less activity and slower action near sacrifice than the control mice; the body weight of the mice in the BLM group showed a clear downward trend during bleomycin-induced pulmonary fibrosis. FK866 (3 mg/kg) significantly reduced BLM-induced weight loss and improved survival rate (Figure 2B-C). Administering 10 mg/kg FK866 similarly reversed BLM-induced weight loss while not causing significant survival improvement (Figure 2B-C), which may due to FK866 reducing NAD levels in some important organs including the heart and liver, and thus lower the mouse survival (Figure S2).

HE staining and Masson's trichrome staining showed that compared with the control, FK866 (10 mg/kg) administration alone caused almost no changes in the lung morphology, while BLM (3 mg/kg) induced significant inflammation and fibrosis in the mouse lung, mainly manifested as destruction of alveolar structure, tissue edema, infiltration of inflammatory cells and deposition of collagen (Figure 2D). Based on the HE staining and Masson's staining, the lung inflammation and fibrosis in the mice were scored using previously reported double-blind, multi-field scoring systems 25, 26. FK866 at 3 mg/kg alleviated BLM-induced lung inflammation and showed a tendency to antagonize the elevated lung injury score (Ashcroft Score). At 10 mg/kg, FK866 significantly mitigated BLM-induced inflammation and collagen deposition in mice lungs (Figure 2D-F).

Meanwhile, FK866 (10 mg/kg) sharply reversed the BLM-induced increment of NAD level in the lung tissue (Figure 2G), while both BLM and FK866 did not alter serum NAMPT levels (Figure 2H). Notably, the baseline level of NAMPT in bronchoalveolar lavage fluid (BALF) was far higher than that in serum. Intratracheal administration of BLM further increased the NAMPT level in the BALF (Figure 2I) but did not affect that in serum (Figure 2H). FK866 (1, 3, 10 mg/kg) significantly decreased the increment of the NAMPT level in BALF of BLM-treated mice (Figure 2I). The results suggested that FK866 might specifically inhibit BLM-induced NAMPT release in the lung.

In addition, FK866 inhibited the release of TNF-α, IL-6, and the classical profibrotic factor TGF-β1 in the BLM-induced fibrotic lungs in mice (Figure 2I). The mRNA levels of four typical collagen factors,* Col1a1*, *Col3a1*, *Tgfb1* and *Acta2*, were increased in mouse lung tissues after bleomycin treatment. FK866 (10 mg/kg) significantly reversed the BLM-induced increase of the collagen factor mRNA synthesis (Figure 2J). The above results indicated that FK866 decreased NAMPT release in the lung and ameliorated bleomycin-induced lung fibrosis injury in mice. Then, we asked which cells in the lung mainly release NAMPT during lung fibrosis.

### NAMPT is highly expressed in lung monocyte/macrophage

**Figure 3:**
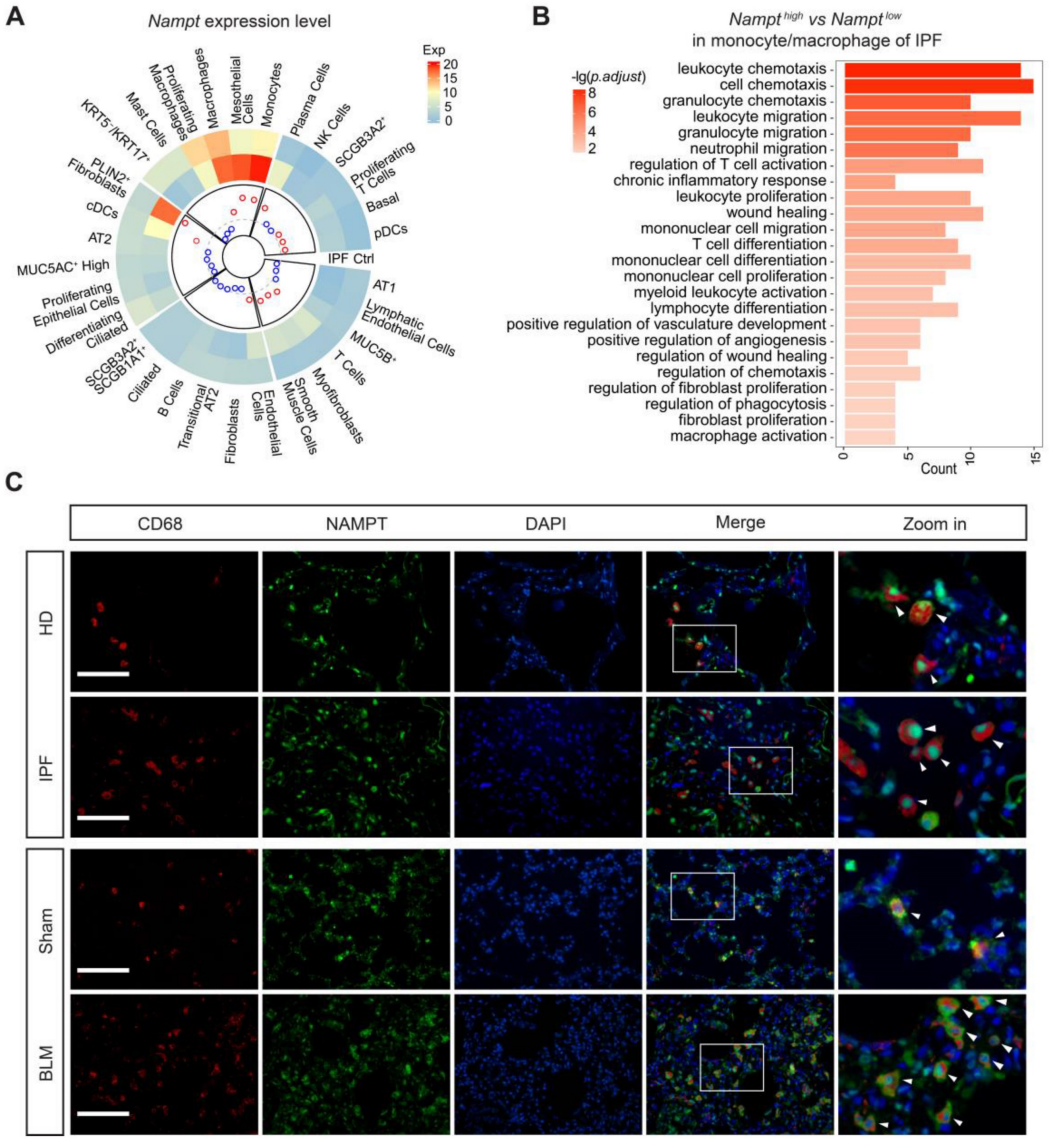
** NAMPT is highly expressed on monocytes/macrophages in fibrotic lungs, which is closely associated with pulmonary fibrosis. (A)** Single-cell transcriptomes of lung cells from 12 IPF and 10 control (Ctrl) were jointly analyzed using the original data from the Banovich/Kropski's dataset (Habermann et al., 2020). The data was annotated according to cell type and diagnosis, and then a circular heatmap was utilized to display NAMPT mRNA expression in each cell type, stratified by disease state. The outer rings represent the log expression level, and the inner rings represent the log fold change (IPF *vs* control) in absolute expression level. The dotted line denotes a fold change of 1 (log 10 FC = 0). The red circles indicate the log 10 FC > 0 and the blue represent the log 10 FC < 0. **(B)** GO enrichment analysis of differentially expressed genes (*Nampt^high^ vs Nampt^low^*) in monocyte/macrophage of IPF. The vertical axis represents the cell signaling pathway category, and the horizontal axis represents the enriched gene count. The color key represents the negative of log 10 (*p*-adjust value). **(C)** Representative images for co-immunostaining of the macrophage marker CD68 (red) and NAMPT (green) in the lung. Bar = 100 μm (n = 3).

Based on the reported single-cell sequencing results (GSE135893) 39, the cells in the lung were identified into 31 different cell populations. We analyzed NAMPT expression levels in these diverse cell populations in both nonfibrotic control and IPF patients (Figure 3A) and determined the cell types mainly responsible for the elevated NAMPT in the lung. It showed that in nonfibrotic controls, macrophages had the highest expression of NAMPT among all identified lung cells (Figure 3A). In IPF patients, the cell populations with high NAMPT expression were monocytes, mesothelial cells, and macrophages (Figure 3A). During lung fibrosis, large numbers of circulating monocytes are recruited into the lung and differentiate into macrophages. Massively infiltrating monocytes and macrophages are critical regulators of pulmonary fibrosis 40, 41. For these NAMPT-expressing monocytes/macrophages, we defined the first 25% of cells as NAMPT-high and the last 25% as NAMPT-low expressing monocytes/macrophages. GO analysis showed that NAMPT high expression-monocytes/macrophages co-expressed more genes associated with fibrosis affairs than NAMPT low expression-monocytes/macrophages did (Figure 3B). Our wet lab data showed that in the lungs of IPF patients and bleomycin-treated mice, CD68-positive (macrophage marker) cell infiltration increased. Furthermore, NAMPT expression increased in the infiltrated macrophages (Figure 3C).

Based on the above results, we then prepared monocyte/macrophage NAMPT-knockout mice and observed the effects of NAMPT-knockout in monocytes/macrophages on bleomycin-induced pulmonary fibrosis in mice.

### Monocyte/macrophage-specific deletion of NAMPT ameliorates BLM-induced pulmonary fibrosis in mice

**Figure 4:**
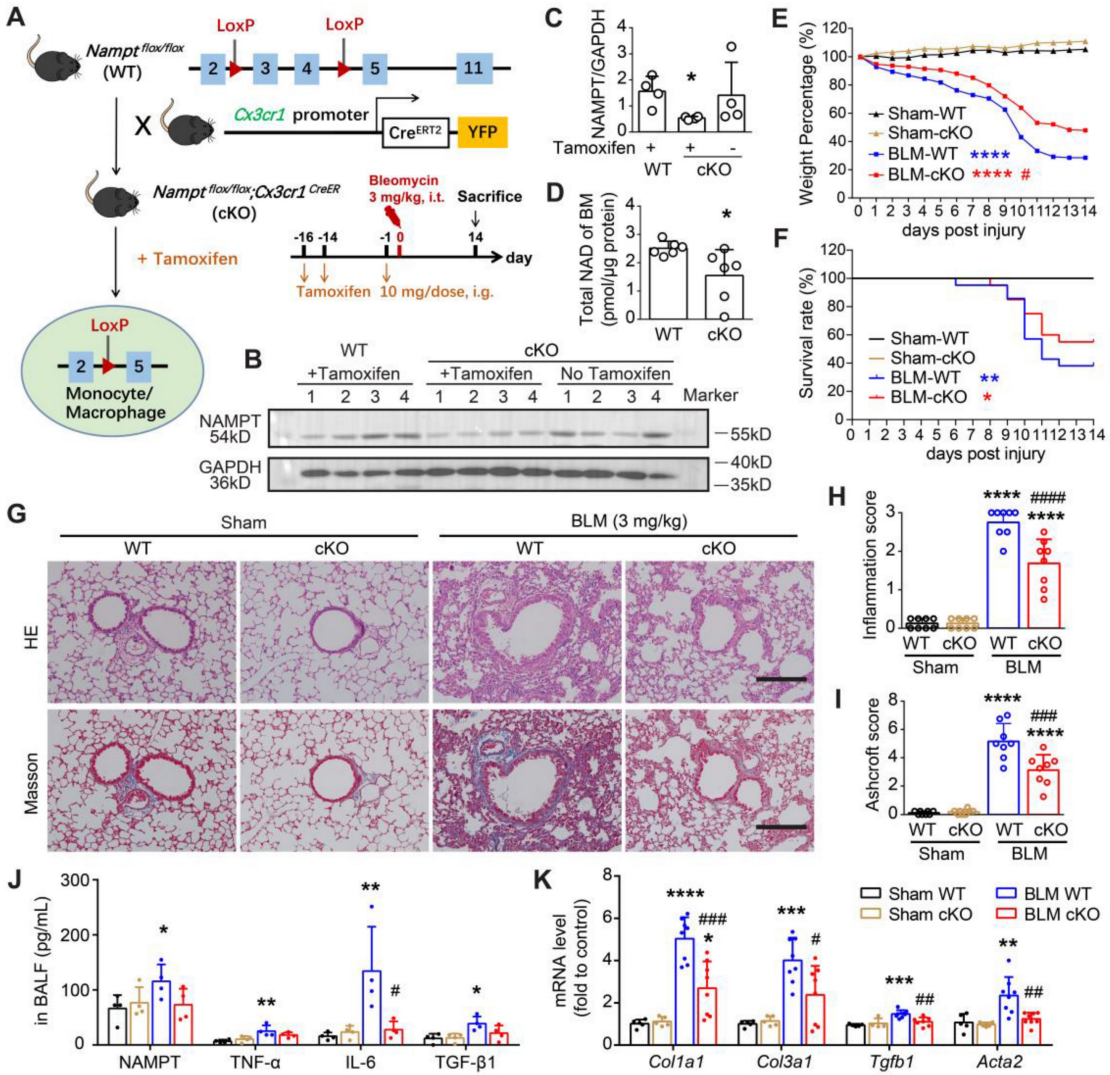
** NAMPT deficiency in monocyte/macrophage ameliorates BLM-induced pulmonary fibrosis in mice. (A)** Schematic draw of the *Nampt* gene conditioning knockout in the monocyte/macrophage of the mouse and the experimental design. **(B, C)** The bone marrow (BM) cells were purified preliminarily by lysing and removing erythrocytes, and then Western blotting analysis was performed to determine the NAMPT expression in the bone marrow cells. Mean ± SD (n = 4), *P < 0.05, compared with WT, unpaired *t*-test. **(D)** Total NAD level of bone marrow cells. Mean ± SD (n = 6), *P < 0.05, unpaired *t*-test. **(E)** Mouse body weight was recorded at the indicated days post lung injury and is shown as percentage of the baseline body weight. **(F)** Kaplan-Meier curves for survival rate of the mice. Sham, n = 12; BLM, n = 20-21. **(G)** Representative HE and Masson's trichrome staining graphs. Bar = 200 μm. **(H, I)** The lung lesions revealed by HE and Masson's trichrome staining were scored double-blindly (n = 8).** (J)** ELISA analysis of eNAMPT, TNF-α, IL-6, and TGF-β1 in BALF (n = 4). **(K)** Relative mRNA expression of collagen factors (*Col1a1*, *Col3a1*, *Tgfb1* and *Acta2*) (n = 5-8). Mean ± SD, *P < 0.05, **P < 0.01, ***P < 0.001, ****P < 0.0001, compared with Sham-WT. *^#^P* < 0.05, *^##^P* < 0.01, *^###^P* < 0.001,*^####^P* < 0.0001, compared with BLM-WT, one-way ANOVA.

CX3CR1 is a commonly used and robust cell surface marker of the monocyte-to-macrophage transition 42. We generated monocyte/macrophage NAMPT conditional knockout mice (cKO mice, genotype: *Nampt^flox/flox^;Cx3cr1^CreER^*) using the Cre/LoxP system. Cre recombinase expression was induced by gavage of tamoxifen (TAM) before bleomycin administration (Figure 4A, Figure S3A). Since Cre recombinase expression ensures CX3CR1-expressing cells express yellow fluorescent protein (YFP) 43, 44, we then collected peripheral blood to detect YFP expression in monocytes (CD45^+^Ly6C^low^) to determine the time course of Cre expression using flow cytometry. It showed that Cre recombination efficiency gradually increased and peaked at two weeks after TAM administration, and then the recombination efficiency decreased (Figure S3B-C). Therefore, we induced Cre expression by gavage of TAM at two weeks and one day before bleomycin administration to ensure that Cre expression was maintained at a high level during the progression of bleomycin-induced lung fibrosis.

Two weeks after TAM administration, the NAMPT expression was significantly reduced in the bone marrow cells of the NAMPT cKO mice (Figure 4B-C). Meanwhile, the NAD level in bone marrow cells isolated from NAMPT cKO mice was significantly decreased (Figure 4D). Immunofluorescence staining showed no Cre expression in lung sections from WT mice, whereas Cre-positive cells were present in lung slices from NAMPT cKO mice (Figure S3D-E). There was co-localization of Cre and F4/80, a marker of macrophages (Figure S3D), whereas no NAMPT expression was observed in Cre-positive cells (Figure S3E). In addition, some of the F4/80-positive macrophages had no NAMPT expression (Figure S3F), further confirming that monocyte/macrophage NAMPT was conditionally silenced. Since CX3CR1 is a marker of the monocyte-to-macrophage transition and also of the lung interstitial macrophages 45, the above results could be interpreted as part of original tissue-resident macrophages and myeloid-derived monocytes that differentiate into macrophages are specifically knocked out of NAMPT.

Then, we induced lung fibrosis in WT and NAMPT cKO mice using BLM. The body weight and survival curves showed conditional knockout of NAMPT in monocytes/macrophages markedly slowed weight loss and improved survival rate in mice with lung fibrosis (Figure 4E-F).

Pathological and biochemical detections showed that conditional knockout of NAMPT in monocytes/macrophages caused no apparent changes in the lungs of sham mice (Figure 4G-K). As HE and Masson's staining showed, NAMPT cKO in monocytes/macrophages significantly mitigated BLM (3 mg/kg)-induced inflammation and fibrosis in the mouse lungs (Figure 4G-I). In addition, the levels of NAMPT, TNF-α, IL-6 and TGF-β1 in BALF and the mRNA levels of *Col1a1*, *Col3a1*, *Tgfb1*, *Acta2* in the lung tissues were detected using ELISA and qRT-PCR, respectively. It showed that NAMPT cKO in monocytes/macrophages decreased the release of these inflammatory factors (Figure 4J) and the synthesis of these collagen factors (Figure 4K) induced by BLM administration in the mice. The above results suggested that NAMPT cKO in monocytes/macrophages significantly ameliorated bleomycin-induced lung fibrosis injury in mice.

### Both deletion and inhibition of NAMPT repress macrophage M2 programming in pulmonary fibrotic mice

NAMPT has been reported to drive macrophage polarization and to be involved in the pathogenesis of many inflammatory diseases 19, 46. To clarify the underlying mechanism of the NAMPT in monocytes/macrophages regulating lung fibrosis injury in mice, we detected macrophage polarization in the lungs of BLM-treated WT and NAMPT cKO mice.

**Figure 5:**
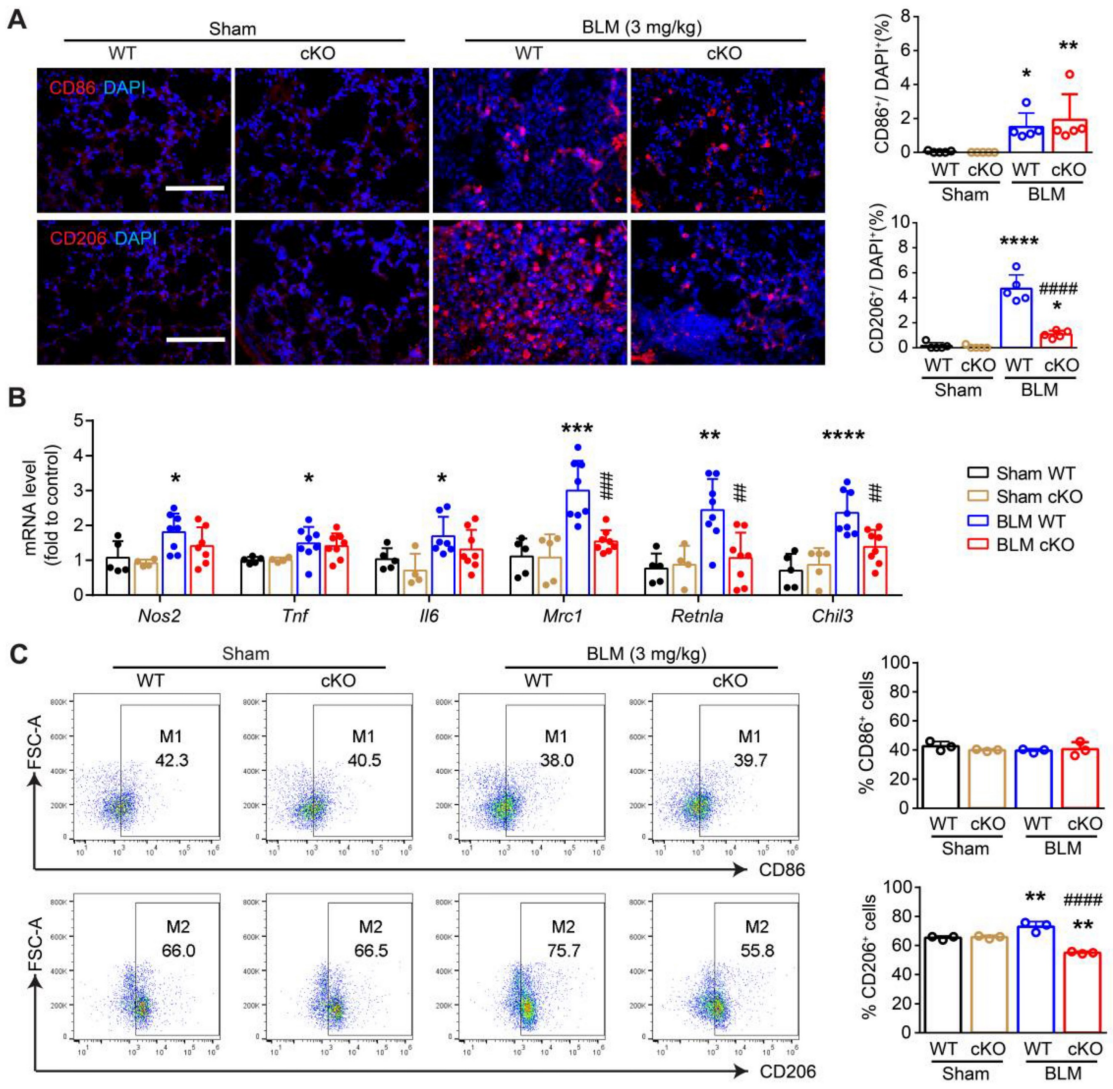
** Deletion of NAMPT in monocytes/macrophages decreases BLM-induced macrophage M2 polarization in mouse lungs.** The mouse with *Nampt* gene conditioning knockout in the monocyte/macrophage, was treated with BLM (3 mg/kg) to induce fibrotic lung injury. Fourteen days after BLM treatment, mice were sampled for lung tissues. **(A)** Immunofluorescence staining of CD86 (M1 marker) or CD206 (M2 marker) in the mouse lungs. Bar = 100 μm (n = 5). **(B)** Relative mRNA expression of M1 markers (*Nos2, Tnf, Il6*) and M2 markers (*Mrc1, Retnla, Chil3*) (n = 5-8). **(C)** Flow cytometry analysis of the proportion of CD86^+^ (M1) and CD206^+^ (M2) macrophages (CD45^+^F4/80^+^CD11b^+^) (n = 3). Mean ± SD. *P < 0.05, **P < 0.01, ***P < 0.001, ****P < 0.0001, compared with Sham-WT. *^##^P* < 0.01, *^###^P* < 0.001, *^####^P* < 0.0001, compared with BLM-WT, one-way ANOVA.

Immunofluorescence staining of CD86 (M1 marker) and CD206 (M2 marker) showed that NAMPT cKO in monocytes/macrophages significantly reduced the proportion of CD206^+^ cells. In contrast, the proportion of CD86^+^ cells showed no trend toward reduction (Figure 5A).

The mRNA levels of M1 markers (*Nos2*, *Tnf,* and *Il6*) and M2 markers (*Mrc1*, *Retnla,* and *Chil3*) were determined using qRT-PCR (Figure 5B). The results showed that NAMPT cKO in monocytes/macrophages strongly inhibited bleomycin-induced differentiation towards M2 macrophages. Meanwhile, there was no significant effect on the mRNA synthesis of M1 markers (Figure 5B). This suggests that NAMPT cKO in monocytes/macrophages mainly affects M2 reprogramming, which was further confirmed by flow cytometry assay (Figure S4, Figure 5C).

**Figure 6:**
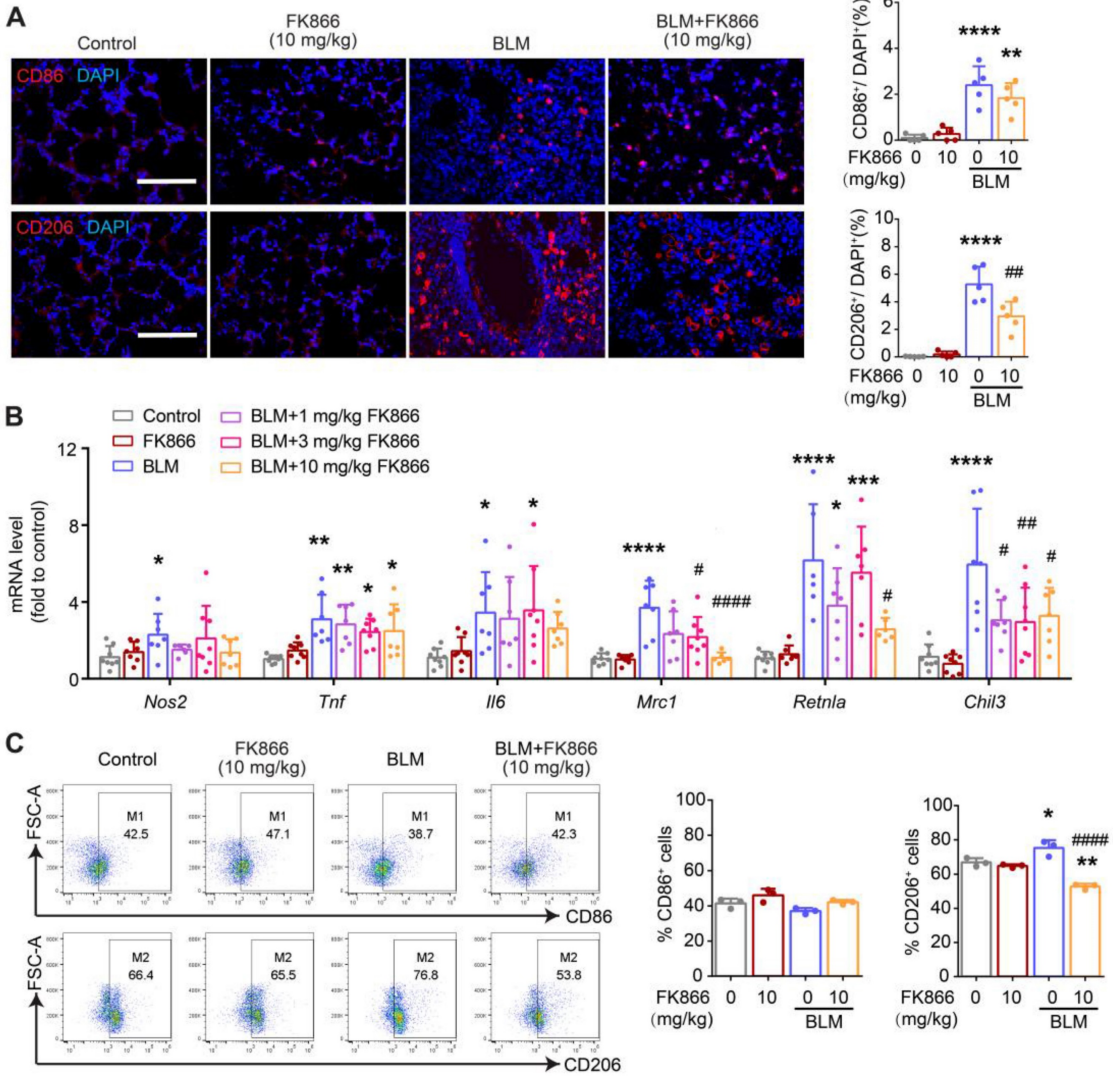
** FK866 decreases bleomycin-induced macrophage M2 polarization in the mouse lung.** Mouse lung fibrosis was induced with one dose of 3 mg/kg bleomycin via intrabronchial injection. FK866 was administrated intraperitoneal injection once per day for 16 consecutive days, starting one day before bleomycin treatment. **(A)** Immunofluorescence staining and analysis of the M1 marker CD86 and the M2 marker CD206 (n = 5). Bar = 100 μm. **(B)** Relative mRNA expression of M1 markers (*Nos2, Tnf, Il6)* and M2 markers (*Mrc1, Retnla, Chil3*) (n = 7-8). **(C)** Flow cytometry analysis for the proportion of CD86^+^ (M1) and CD206^+^ (M2) among lung macrophages (CD45^+^F4/80^+^CD11b^+^) (n = 3). Mean ± SD. *P < 0.05, **P < 0.01, ***P < 0.001, ****P < 0.0001, compared with Control. *^#^P* < 0.05, *^##^P* < 0.01, *^####^P* < 0.0001, compared with bleomycin treatment (BLM), one-way ANOVA.

Similarly, FK866 at 10 mg/kg showed no apparent effect on BLM-induced CD86 (M1 marker) expression but inhibited BLM-induced CD206 (M2 marker) expression (Figure 6A). In addition, FK866 (1, 3, 10 mg/kg) significantly decreased BLM-induced upregulation of *Mrc1, Retnla*, and* Chil3* (M2 markers) mRNA levels. Still, it showed no effects on the mRNA level of *Nos2, Tnf,* and* Il6* (M1 markers) in the fibrotic lungs of BLM-treated mice (Figure 6B). Flow cytometry assay showed that FK866 at 10 mg/kg inhibited BLM-induced increment of CD206^+^ macrophages in the mouse lungs (Figure 6C).

These results suggested that both NAMPT cKO in monocytes/macrophages and NAMPT inhibitor FK866 mitigated BLM-induced lung fibrosis injury in mice, at least partly through inhibiting macrophages M2 programming.

### NAMPT prompts pulmonary fibrosis by promoting macrophage M2 polarization

**Figure 7:**
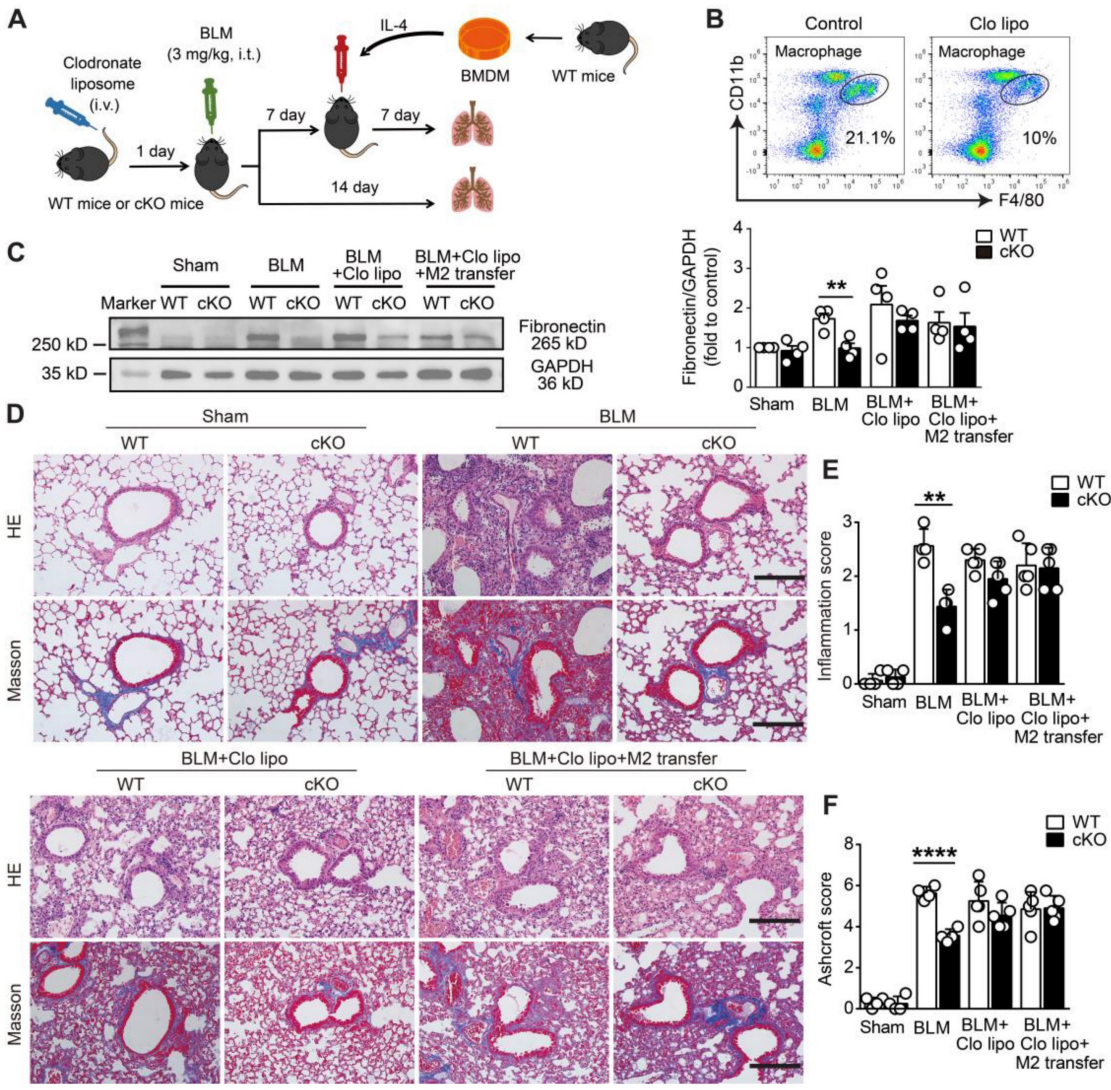
** Deletion of NAMPT in monocytes/macrophages protects mice against BLM-induced pulmonary fibrosis by reducing M2 programming. (A)** Schematic representation of the macrophage adoptive transfer experiment design. Bone marrow-derived macrophages (BMDMs) from WT mice were induced to M2 polarization using IL-4 (20 ng/mL). The IL-4-induced WT M2 BMDMs were adoptively transferred into clodronate liposome-treated or control liposome-treated WT and NAMPT cKO mice (specific deletion of NAMPT in monocyte/macrophage) through intratracheal injection seven days after BLM administration. **(B)** Flow cytometry analysis for the proportion of macrophages 24 h after the tail vein injection of clodronate liposome (n = 3). **(C)** Western blot analysis of Fibronectin protein level in the mouse lungs (n = 4). **(D)** Representative graphs of HE and Masson's trichrome staining. Bar = 200 μm. **(E, F)** Lung inflammation and fibrosis score (n = 4-5). Mean ± SD, **P < 0.01, ****P < 0.0001, unpaired *t*-test.

To further confirm the protective effect of monocyte/macrophage NAMPT cKO or FK866 against lung fibrosis depending on the inhibition of M2 polarization, a macrophage transfer experiment was performed in BLM-induced lung fibrosis mice. First, clodronate liposomes were injected via the tail vein to exhaust monocytes/macrophages in the peripheral blood of WT and NAMPT cKO mice (Figure 7A). One day after macrophage depletion (Figure 7B), mice were injected intratracheally with BLM (3 mg/kg) to induce lung fibrotic injury. Western blotting assay showed BLM-treatment caused significant fibrotic injury in WT mice, NAMPT cKO reversed the increased fibronectin levels (Figure 7C). HE and Masson's trichrome staining showed that NAMPT cKO alleviated the inflammation and tissue destruction induced by BLM-treatment in mice (Figure 7D).

Then, M2 macrophage replenishment was performed 7 days after BLM treatment. Briefly, BMDMs isolated from WT mice were induced into M2 macrophages by IL-4 (20 ng/mL). The prepared WT M2 macrophages were adoptively transferred via intratracheal injection into clodronate liposome-treated WT and cKO mice, where the macrophages were depleted (Figure 7A). As expected, when the mice were subjected to BLM-induced fibrotic lung injury, adoptive transfer of WT M2 macrophages significantly reversed the protective effect of monocyte/macrophage *Nampt* deficiency in NAMPT cKO mice, which showed fibrotic marker levels and lung injury scores similar to WT mice did (Figure 7C-F). These results further indicated that NAMPT prompted pulmonary fibrosis by promoting macrophage M2 polarization.

### NAMPT elevated in macrophages under TGF-β1-induced fibrotic microenvironment, accompanied with M2 polarization

The above *in vivo* experiments strongly suggested that NAMPT drives pulmonary fibrosis injury in mice by promoting macrophage M2 polarization. To elucidate the mechanism underlying NAMPT promotion of M2 polarization, the *in vitro* experiments were performed in murine RAW264.7 macrophages, where TGF-β1 was used to mimic the fibrotic microenvironment, FK866 was used to inhibit NAMPT activity, and NAMPT siRNA was used to decrease NAMPT expression.

**Figure 8:**
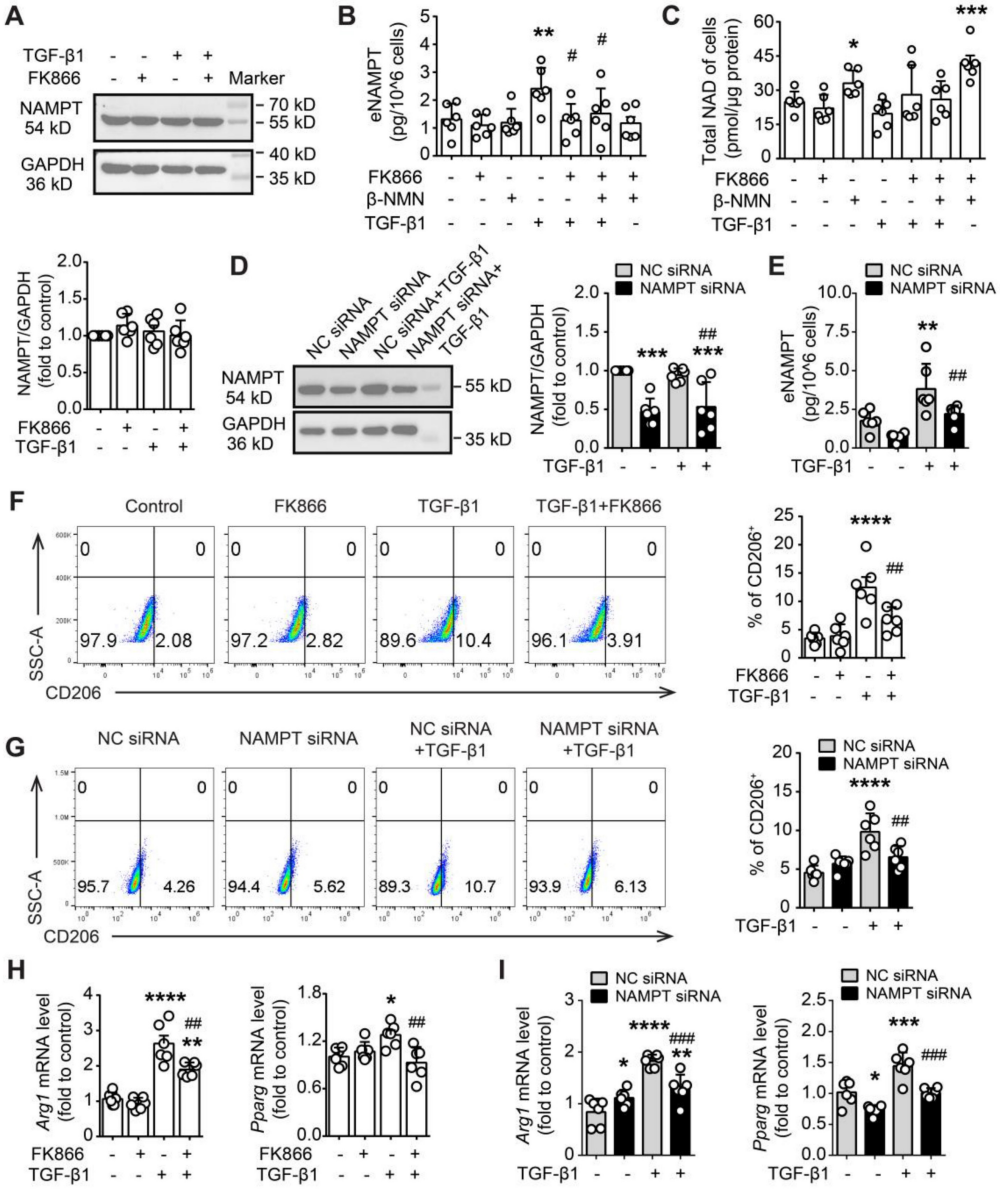
** FK866 and NAMPT siRNA inhibit TGF-β1-induced NAMPT releasing and M2 polarization in RAW264.7 macrophages. (A-C, F, H)** RAW264.7 cells were treated with FK866 (1 nM) and β-NMN (100 μΜ) 30 min before the addition of TGF-β1 (20 ng/mL). **(D-E, G, I)** RAW264.7 cells were transfected with negative control (NC) siRNA or NAMPT siRNA, and then treated with TGF-β1 (20 ng/mL) for 24 h. **(A, D)** Western blot analysis of NAMPT expression. **(B, E)** ELISA assay of NAMPT releasing in the culture supernatant. **(C)** Quantification of the intracellular NAD level using a commercial kit. **(F, G)** Flow cytometry analysis of CD206-positive cells (M2 polarization). **(H, I)** qRT-PCR analysis of M2 polarization markers (*Arg1* and* Pparg*). Mean ± SD (n = 6). *P < 0.05, **P < 0.01, ***P < 0.001, ****P < 0.0001, compared with Control or NC siRNA, *^#^P* < 0.05, *^##^P* < 0.01, *^###^P* < 0.001, compared with TGF-β1 or NC siRNA+ TGF-β1, one-way ANOVA.

TGF-β1 (20 ng/mL) showed almost no effect on NAMPT expression (Figure 8A), whereas it significantly increased NAMPT release in RAW264.7 cells (Figure 8B). Pre-treatment with FK866 (1 nM) for 30 min reversed the increment of NAMPT releasing induced by TGF-β1 stimulation for 24 h (Figure 8B, Figure S5A). Intriguingly, NAD precursor β-NMN (100 μM) did not affect TGF-β1-induced NAMPT releasing (Figure 8B, Figure S5B), though β-NMN significantly increased the intracellular NAD level (Figure 8C). In addition, both FK866 (1 nM) and TGF-β1 (20 ng/mL) showed no effects on the intracellular NAD level (Figure 8C). The above results suggested that NAMPT release and FK866 inhibition of TGF-β1-induced NAMPT release were independent of intracellular NAD levels.

Then, we supposed that the release of NAMPT might be affected by the intracellular NAMPT expression level. Indeed, NAMPT siRNA significantly reduced TGF-β1-induced NAMPT release (Figure 8D-E). Furthermore, FK866 and NAMPT siRNA decreased TGF-β1-induced M2 polarization in RAW264.7, including a decrease in the proportion of CD206-positive cells and downregulation of mRNA levels of M2 marker (*Arg1* and *Pparg*) (Figure 8F-I).

Overall, these results suggested that TGF-β1 induced the release of intracellular NAMPT into the extracellular space, and then the extracellular NAMPT promoted M2 polarization in RAW264.7 cells.

### Extracellular NAMPT enzymatic-independently induces M2 polarization and TGF-β1 release in RAW264.7 cells

**Figure 9:**
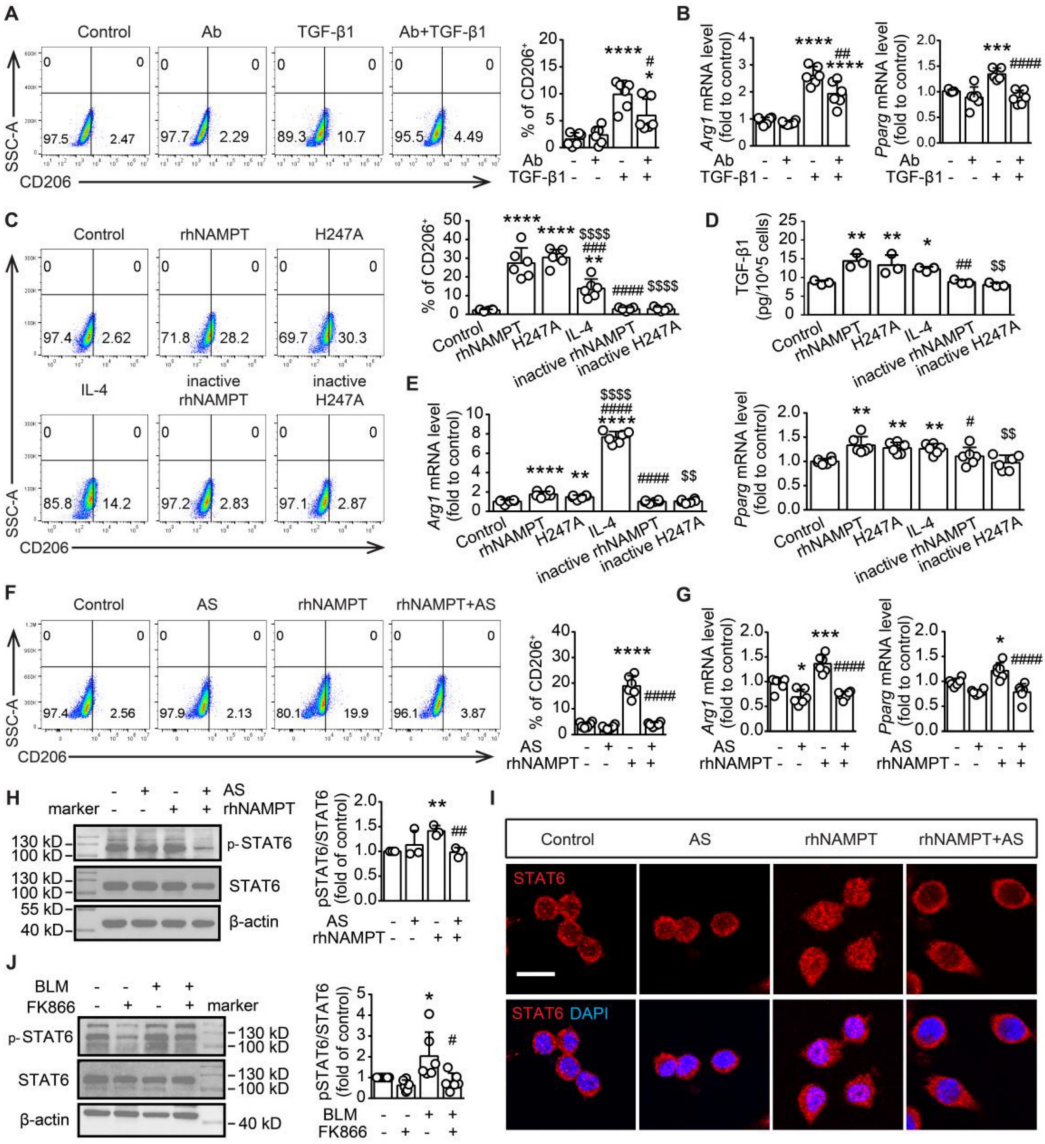
** Extracellular NAMPT induces M2 polarization in an enzyme-independent manner by activating the STAT6 pathway. (A-B)** RAW264.7 were exposed to TGF-β1 (20 ng/mL) for 24 h, with or without pre-treatment with NAMPT antibody (Ab, 0.55 ng/mL) to neutralize the released eNAMPT, and then subjected to flow cytometry analysis of M2 programming (CD206 as a marker) (A), and mRNA level detection of M2 polarization markers (*Arg1, Pparg*) (B). **(C-E)** RAW264.7 were treated with PBS (vehicle), rhNAMPT (300 nM), NAMPT^H247A^ (H247A, 300 nM), IL-4 (30 ng/mL), heat-inactive NAMPT protein (300 nM) or heat-inactive NAMPT^H247A^ (300 nM) for 24 h, and fed to analysis of M2 polarization (C, E) and TGF-β1 releasing (D). **(F-I)** RAW264.7 cells were treated with a STAT6 inhibitor AS1517499 (AS, 10 nM) for 1 h, followed by rhNAMPT (300 nM) stimulation for 24 h, and then subjected to flow cytometry analysis (F), mRNA level detection (G), quantification of pSTAT6/STAT6 ratio (H), immunofluorescence of STAT6 (red) and DAPI (blue) to show STAT6 nuclear translocation (I). Bar = 20 μm. **(J)** Mouse lung fibrosis was induced with one dose of 3 mg/kg bleomycin via intrabronchial injection. FK866 (10 mg/kg) was administrated by intraperitoneal injection once per day for 16 consecutive days starting one day before bleomycin treatment. The protein level of STAT6 and phosphor-STAT6 were determined using Western blotting. Mean ± SD (n = 3-6). *P < 0.05, **P < 0.01, ***P < 0.001, ****P < 0.0001, compared with Control. *^#^P* < 0.05, *^##^P* < 0.01, *^####^P* < 0.0001, compared with TGF-β1 or rhNAMPT or BLM, one-way ANOVA (A-B, F-J). *P < 0.05, **P < 0.01, ****P < 0.0001, compared with Control. *^#^P* < 0.05, *^##^P* < 0.01, *^###^P* < 0.001, *^####^P* < 0.0001, compared with rhNAMPT. *^$^P* < 0.05, *^$$^P* < 0.01, *^$$$$^P* < 0.0001, compared with H247A, one-way ANOVA (C-E).

To confirm the released extracellular NAMPT promotes M2 polarization in RAW264.7 macrophages under the TGF-β1-induced fibrotic microenvironment, we employed NAMPT antibody (NAMPT Ab) to neutralize the released eNAMPT during TGF-β1 treatment in RAW264.7 cells (Figure 9A-B). The concentration of NAMPT Ab (0.55 ng/mL) was applied based on the previous measurement of eNAMPT in the supernatant of RAW264.7 cells stimulated with TGF-β1 (Figure 8B). The selected concentration was more than ten times the eNAMPT level in the supernatant. Flow cytometry and qRT-PCR analysis revealed a significant downregulation of macrophage M2 polarization upon neutralization of eNAMPT using NAMPT Ab (Figure 9A-B). Furthermore, home-made recombinant human NAMPT protein (rhNAMPT) 27 at a concentration of 300 nM was used to mimic the effect of eNAMPT, with heat-inactive NAMPT protein as a negative control and IL-4 as a positive control. To our surprise, rhNAMPT, like IL-4, induced M2 polarization (Figure 9C-E).

In addition, to address whether the role of eNAMPT in inducing macrophage polarization is related to its enzymatic activity, the NAMPT mutant protein NAMPT^H247A^ with almost no enzymatic activity 47 was used. Similar to wild-type NAMPT protein, NAMPT^H247A^ (300 nM) increased CD206^+^ cells as well as mRNA expression of *Arg1* and *Pparg* (Figure 9C, E). Notably, both rhNAMPT and NAMPT^H247A^ markedly induced TGF-β1 release in RAW264.7 cells (Figure 9D). The results suggested that the induction of M2 polarization by eNAMPT is independent of its enzymatic activity.

### Extracellular NAMPT induces M2 polarization through STAT6 activation in RAW264.7 cells

To explore the signaling pathway involved in the induction of M2 polarization by extracellular NAMPT, lung tissues from BLM-treated WT and NAMPT cKO mice were used for RNA-seq. KEGG analysis revealed the enriched relative cell signaling pathway with significant P value, including JAK-STAT signaling pathway which was closely associated with macrophage polarization (Figure S6). STAT6 is known to drive M2 polarization 48. Thus, rhNAMPT was used to mimic extracellular NAMPT, and the STAT6 pathway inhibitor AS1517499 was applied (Figure S5C). AS1517499 (10 nM) decreased rhNAMPT-induced increment of CD206-positive cells and mRNA level of M2 markers (*Arg1* and *Pparg*) (Figure 9F-G). Moreover, rhNAMPT (300 nM) treatment for 24 h increased STAT6 phosphorylation (Figure 9H) and promoted STAT6 nuclear translocation (Figure 9I), which was reversed by AS1517499. Notably, we also observed FK866 inhibiting the STAT6 pathway activation in BLM-induced mouse fibrotic lungs (Figure 9J). As such, the above results indicated that eNAMPT mediates macrophage M2 programming partly by activating STAT6 pathway.

## Discussion

Pulmonary fibrosis is a progressive chronic interstitial lung disease with high mortality. The treatment of this disease remains a significant clinical challenge due to its complex pathogenesis mechanisms. In this study, we found that the levels of NAMPT were upregulated in the fibrotic lung tissues, and that the release of NAMPT by macrophages increased. The extracellular NAMPT promoted M2 polarization of macrophages and the release of TGF-β1, thus promoting pulmonary fibrosis. NAMPT inhibition in macrophages might be a novel approach for preventing and treating pulmonary fibrosis.

NAMPT is suggested to serve as a biomarker for pulmonary fibrosis 49. However, the role of NAMPT in pulmonary fibrosis is controversial. It has shown that NAMPT from damaged lung epithelial cells promotes radiation-induced pulmonary fibrosis 50, 51 and that NAMPT temporally reinforces pro-fibrotic myofibroblast phenotypes in age-dependent pathological fibrosis 52, while another study 53 using mesenchymal stem cells co-cultured with alveolar type II epithelial cells reported that upregulation of NAMPT expression and NAD^+^ levels in mesenchymal stem cells can slow down the aging of alveolar type II epithelial cells, thus exerting protective effects against lung fibrosis. This controversy can be explained by the fact that NAMPT exerts diverse effects in different cells through both intracellular and extracellular forms under specific conditions. Extracellular NAMPT has been reported to act mainly as a damage-associated molecular pattern, mediating and exacerbating liver and heart fibrosis 50, 54, 55. At the same time, iNAMPT shows enzymatic effects on maintaining or increasing NAD^+^ levels to protect organs 56. Here, our *in vivo* and *in vitro* experiments confirmed that during the process of pulmonary fibrosis, NAMPT expression was upregulated, and NAMPT release was increased in the lung, promoting the progression of pulmonary fibrosis.

We found that NAMPT inhibitor FK866 alleviated BLM-induced lung fibrotic injury by inhibiting NAMPT release and macrophage M2 programming. FK866 previously showed anti-fibrosis and antioxidant effects through maintenance of NAD^+^ homeostasis during diabetic nephropathy 56, while NAMPT inhibitors were developed for treating cancer, their anti-cancer effects are dose-dependent. Generally, administrating FK866 at 10 - 40 mg/kg per day 57 in mice significantly inhibited NAD production, exerting anti-cancer effects. However, the dose-limiting toxicities of NAMPT inhibitors, such as thrombocytopenia, have led to the discontinuation in phase I/II clinical trials 58. In this study, we found that FK866 at a high dose (10 mg/kg) significantly improved BLM-induced inflammation and fibrotic injury in the lung, but did not improve the survival rate of the mice. Meanwhile, FK866 (10 mg/kg) decreased NAD levels in the lung, heart and liver, which suggested that NAMPT inhibitor exhibited dose-dependent toxicity.

In addition to traditional chemical drug therapy, gene editing and cell therapy have been gradually developed in recent years. Since *Nampt* homozygous knockout mice (NAMPT^-/-^) have embryonic lethality 59, many studies have to choose heterozygous mice (NAMPT^+/-^) to mimic the downregulation of NAMPT systemically 50. In this study, we analyzed the publicly available single-cell transcriptome database of lung fibrosis patients. We found that the level of NAMPT is different in individual cell types, with the highest expression levels in lung monocytes and macrophages. In contrast, many cells including AT2 cells, downregulate NAMPT expression during pulmonary fibrosis. To minimize the side effects of the systemic intervention on the NAMPT levels, we prepared the mouse with conditional knockout of NAMPT in monocytes/macrophages.

Macrophages are distributed throughout the lung and can be broadly divided into alveolar macrophages (AM), located in the airways/alveoli, and interstitial macrophages (IM), presented in the interstitial/parenchyma of the lung. AMs maintain their numbers by self-renewal in a steady-state environment, while IMs in adult mice are derived from blood monocytes 60. Following tissue injury, many resident macrophages die, and the lung parenchyma initiates an innate immune response characterized by the differentiation of bone marrow-derived monocytes to supplement the macrophage pool 61. In the context of pulmonary fibrosis, monocyte-derived macrophages outnumber resident macrophages and participate in inflammation, fibrosis progression or regression at different stages of pulmonary fibrosis 62. Here, we found that conditional knockout of NAMPT in monocytes/macrophages (NAMPT cKO) corrected the dysregulated polarization of infiltrating macrophages, significantly improving lung fibrosis damage and survival in mice.

Single-cell technology has developed rapidly in recent years, allowing for a more detailed classification of macrophages 63. In this study, we continued the M1/M2 classification method from a functional perspective, with M1 referring to pro-inflammatory and anti-fibrotic states and M2 referring to anti-inflammatory and pro-fibrotic states 64. NAMPT has been reported to regulate macrophage polarization, but its bias towards macrophage polarization varies in different disease states 65. We found that the specific deletion of NAMPT in monocytes/macrophages inhibited M2 polarization and alleviated BLM-induced pulmonary fibrosis in mice, whereas supplementation of M2 macrophages restored the susceptibility of mice to BLM-induced injury. To our knowledge, we, for the first time, confirm that NAMPT is an essential mediator in regulating the transformation of monocytes/macrophages to pro-fibrotic phenotype in the process of pulmonary fibrosis.

Furthermore, we clarified that extracellular NAMPT drives macrophage M2 polarization and promotes pulmonary fibrosis. TGF-β1 is a promoter molecule of pulmonary fibrosis and a hallmark of fibrotic effector molecules 66. We found that TGF-β1 stimulates macrophages to release eNAMPT and, conversely, that rhNAMPT protein can promote TGF-β1 release in a non-enzymatic dependent manner. The above is similar to what we have previously seen in conditions of cerebral ischemic injury. Inflammatory factors such as TNF-α can promote the release of NAMPT by microglia, the monocyte/macrophage in the central nervous system, and NAMPT can also promote the release of TNF-α by microglia 67, 68. Similarly, FK866 mitigates cerebral ischemia-reperfusion injury 67. In this study, eNAMPT and TGF-β1 were detected in the alveolar lavage fluid of the pulmonary fibrosis mice. Based on the previous report that BLM-injury promotes the synthesis and release of TGF-β1 by lung epithelial cells 69, as well as our *in vitro* results, it shows that the released TGF-β1 induces macrophages to synthesize and release NAMPT, and that these autocrine and paracrine eNAMPT further promote infiltrated macrophage M2 polarization and TGF-β1 secretion, which leads to and exacerbates macrophage polarization imbalance, strengthens the fibrotic microenvironment, and promotes disease progression.

It was speculated that eNAMPT exerts its effects by acting on the corresponding receptors on the cell membrane. In line with previous reports 70, 71, we pretreated macrophages with C-C chemokine receptor type 5 (CCR5) and Toll-like receptor 4 (TLR4) blockers, which indeed partially alleviated rhNAMPT-induced M2 polarization in macrophages (Figure S7). However, in the central nervous system, microglia release NAMPT into the extracellular space in exosome and 'nude' form 68, which indicates that the eNAMPT exerts its effects not only via corresponding receptors. Further research is needed to elucidate the way that eNAMPT acts on cells.

Our RNA-seq analysis on NAMPT cKO and WT mice lung with BLM-induced fibrotic injury revealed significant changes in the PI3K/Akt, JAK-STAT signaling pathway. Though several signaling involved in M2 polarization including JAK/STAT, PI3K/Akt, and AMPK pathway, activation of signal transducer and activator of transcription (STAT) is the key signaling required for macrophage polarization 72, 73. Here, we ultimately confirmed that in BLM-induced mouse pulmonary fibrosis, NAMPT at least partially promotes macrophage M2 polarization by activating STAT6, which was consist with the previous report that STAT6 mediated M2 programing 74.

This study has limitations. Here, we focus on the effect of eNAMPT on pulmonary fibrosis, emphasizing its impact on macrophage M2 polarization. However, the interaction or transformation between eNAMPT and iNAMPT is still unknown. In addition, whether iNAMPT has other roles in this process, such as regulating cell energy metabolism and affecting macrophage polarization to participate in the pathogenesis of pulmonary fibrosis, remains to be answered.

In summary, our study revealed that NAMPT prompts pulmonary fibrosis by driving macrophage M2 polarization in an enzymatic-independent, STAT6-dependent manner; furthermore, our study paves the way to develop novel anti-pulmonary fibrosis strategies based on deletion of NAMPT in macrophage.

## Supplementary Material

Supplementary figures.

## Figures and Tables

**Table 1:** Mouse-specific primer pairs used for qRT-PCR experiments.

Name	Sequences (5′ to 3′)
*Actb*	F: GGCTGTATTCCCCTCCATCG
	R: CCAGTTGGTAACAATGCCATGT
*Nampt*	F: GCAGAAGCCGAGTTCAACATC
	R: TTTTCACGGCATTCAAAGTAGGA
*Col1a1*	F: GCTCCTCTTAGGGGCCACT
	R: CCACGTCTCACCATTGGGG
*Col3a1*	F: CTGTAACATGGAAACTGGGGAAA
	R: CCATAGCTGAACTGAAAACCACC
*Tgfb1*	F: CTCCCGTGGCTTCTAGTGC
	R: GCCTTAGTTTGGACAGGATCTG
*Acta2*	F: GGACGTACAACTGGTATTGTGC
	R: TCGGCAGTAGTCACGAAGGA
*Retnla*	F: CCAATCCAGCTAACTATCCCTCC
	R: ACCCAGTAGCAGTCATCCCA
*Chil3*	F: CAGGTCTGGCAATTCTTCTGAA
	R: GTCTTGCTCATGTGTGTAAGTGA
*Mrc1*	F: CTCTGTTCAGCTATTGGACGC
	R: CGGAATTTCTGGGATTCAGCTTC
*Tnf*	F: CCTGTAGCCCACGTCGTAG
	R: GGGAGTAGACAAGGTACAACCC
*Il6*	F: CTGCAAGAGACTTCCATCCAG
	R: AGTGGTATAGACAGGTCTGTTGG
*Nos2*	F: GGATCTTCCCAGGCAACCA
	R: CAATCCACAACTCGCTCCAA
*Arg1*	F: CTCCAAGCCAAAGTCCTTAGAG
	R: AGGAGCTGTCATTAGGGACATC
*Pparg*	F: GGAGCCTAAGTTTGAGTTTGCTGTG
	R: TGCAGCAGGTTGTCTTGGATG

## Abbreviations

Ab: antibody
rhNAMPT: recombinant human NAMPT protein
AM: alveolar macrophage
BALF: bronchoalveolar lavage fluid
BLM: bleomycin
BM: bone marrow
BMDM: bone marrow-derived macrophages
CCR5: C-C chemokine receptor type 5
cKO: conditional knockout
DEG: differentially expressed gene
ELISA: Enzyme linked immunosorbent assay
eNAMPT: extracellular NAMPT
FC: fold change
GO: gene ontology
HD: healthy donor
IH: Immunohistochemistry
IM: interstitial macrophage
iNAMPT: intracellular NAMPT
IPF: idiopathic pulmonary fibrosis
KEGG: Kyoto Encyclopedia of Genes and Genomes
NAD: nicotinamide adenine dinucleotide
NAMPT: nicotinamide phosphoribosyltransferase
PBEF: pre-B-cell clonogenic enhancer factor
RNA-Seq: RNA Sequencing
TAM: tamoxifen
TLR4: Toll-like receptor 4
WT: wild-type
YFP: yellow fluorescent protein
