# Supplementary material for: Nicotinamide phosphoribosyltransferase prompts bleomycin-induced pulmonary fibrosis by driving macrophage M2 polarization in mice
Source: Theranostics. 2024 Apr 28;14(7):2794–815. doi: 10.7150/thno.94482 (PMC11103509; doi:10.7150/thno.94482)
Supplement: Supplementary file 1 — Supplementary figures. [file thnov14p2794s1.pdf]

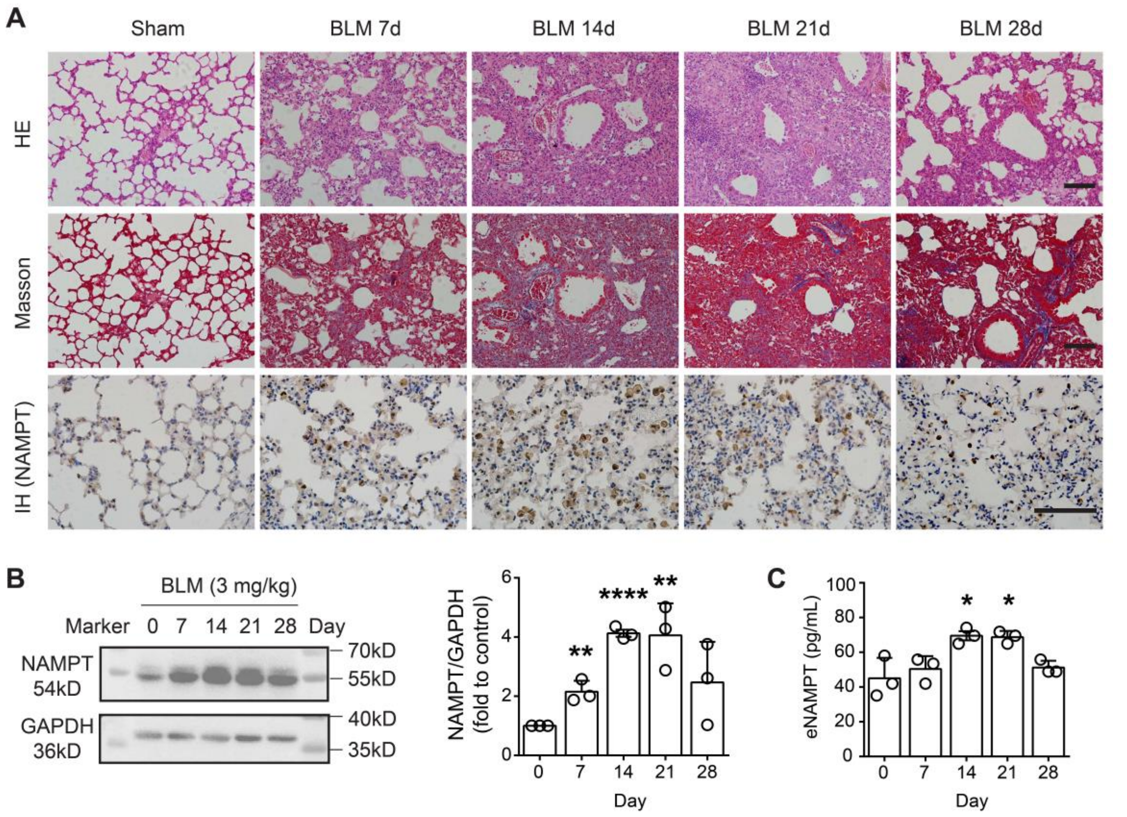

Figure S1 NAMPT level changes in the mouse lungs during BLM-induced pulmonary fibrosis.

(A) Representative graphs of HE, Masson's trichrome staining, and immunohistochemical staining of NAMPT in mouse lungs. Bar = 100 μm. (B) Western blotting analysis and quantification for the expression of NAMPT in the lungs of mice. (C) ELISA analysis of extracellular NAMPT in BALF. Mean ± SD (n = 3), *P < 0.05, **P < 0.01, ****P < 0.0001, compared with Sham, unpaired t-test.

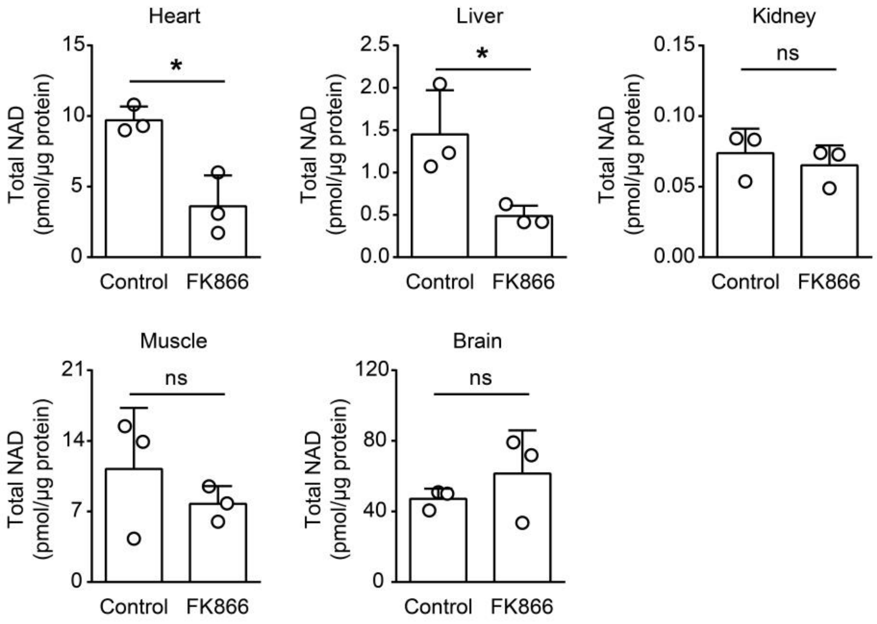

Figure S2 Effect of FK866 on the NAD level in mouse tissues.
Saline (Control) or 10 mg/kg FK866 (FK866) was administered once per day via intraperitoneal injection for 16 consecutive days. After saline rinsing, mouse tissues (heart, liver, kidney, muscle, and brain) were harvested, homogenized, and subjected to NAD determination using a commercial kit. Mean ± SD (n = 3). *$P < 0.05$, unpaired *t*-test.

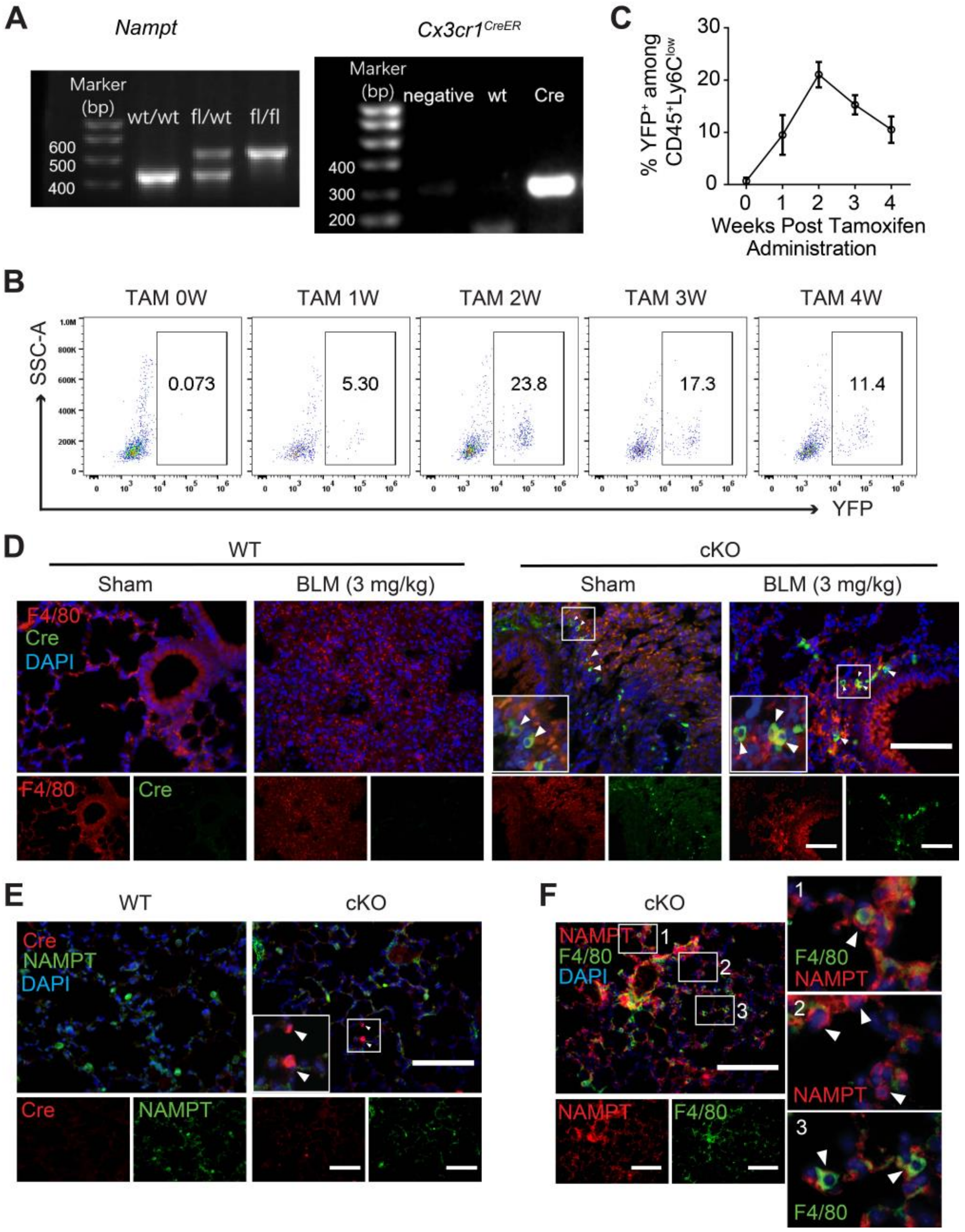

Figure S3 Verification of specific knockout of NAMPT in monocytes/macrophages. (A) Gene identification of transgenic mice. (B, C) Time course of Cre expression after tamoxifen administration by detecting the expression of the YFP reporter in peripheral blood monocytes ($CD45^{+}Ly6C^{low}$). Mean ± SD (n = 3 mice per timepoint). (D) Immunofluorescence staining of macrophage marker F4/80 (red) and Cre (green) in the lungs from control and NAMPT cKO mice. (E) Immunofluorescence staining of Cre (red) and NAMPT (green) in the lung sections. (F) Immunofluorescence staining of NAMPT (red) and F4/80 (green) in the lung of cKO mice. Bar = 100 μm (n = 3).

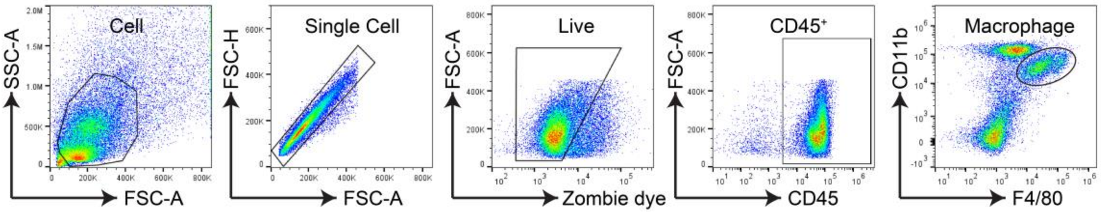

Figure S4 Sequential gating strategy to identify lung macrophage subsets by flow cytometry analysis.

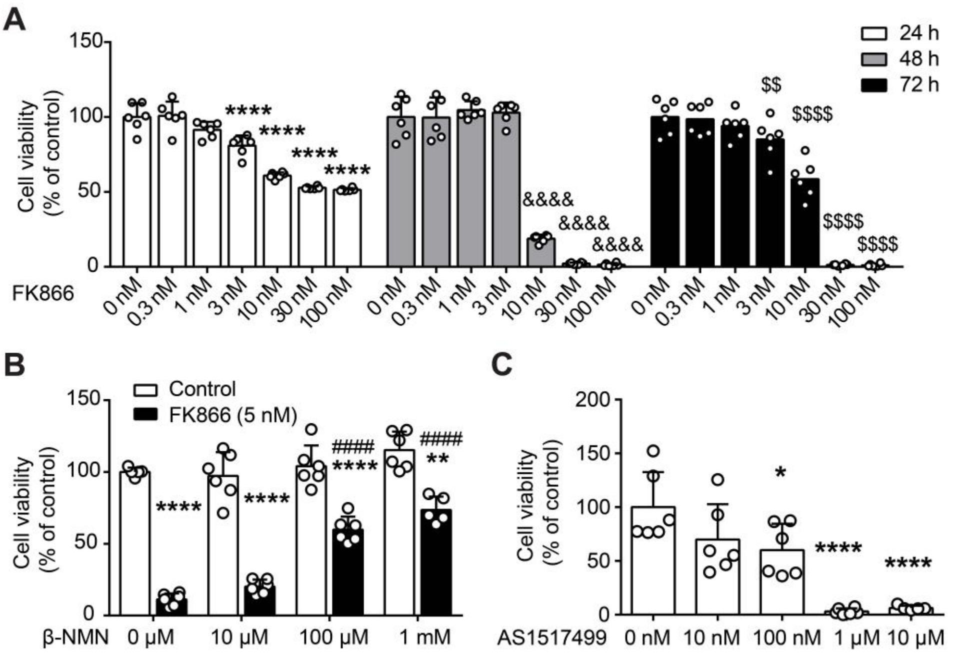

Figure S5 Effects of FK866, β-NMN, AS1517499 on the viability in RAW264.7 cells. RAW264.7 cells were treated with FK866 for 24 h, 48 h, 72 h or β-NMN, AS1517499 for 24 h, and then CCK8 was added. The OD_{450} was detected 2 h later. Mean ± SD (n = 6). $^{****}P < 0.0001$, compared with 24 h-0 nM FK866; $^{\&\&\&\&}P < 0.0001$, compared with 48 h-0 nM FK866; $^{\$\$}P < 0.01$, $^{\$\$\$\$}P < 0.0001$, compared with 72 h-0 nM FK866 (A); $^{**}P < 0.01$, $^{****}P < 0.0001$, compared with Control + 0 μM β-NMN; $^{\#\#\#\#}P < 0.0001$, compared with FK866 + 0 μM β-NMN (B); $^{*}P < 0.05$, $^{****}P < 0.0001$, compared with 0 nM AS1517499 (C); one-way ANOVA.

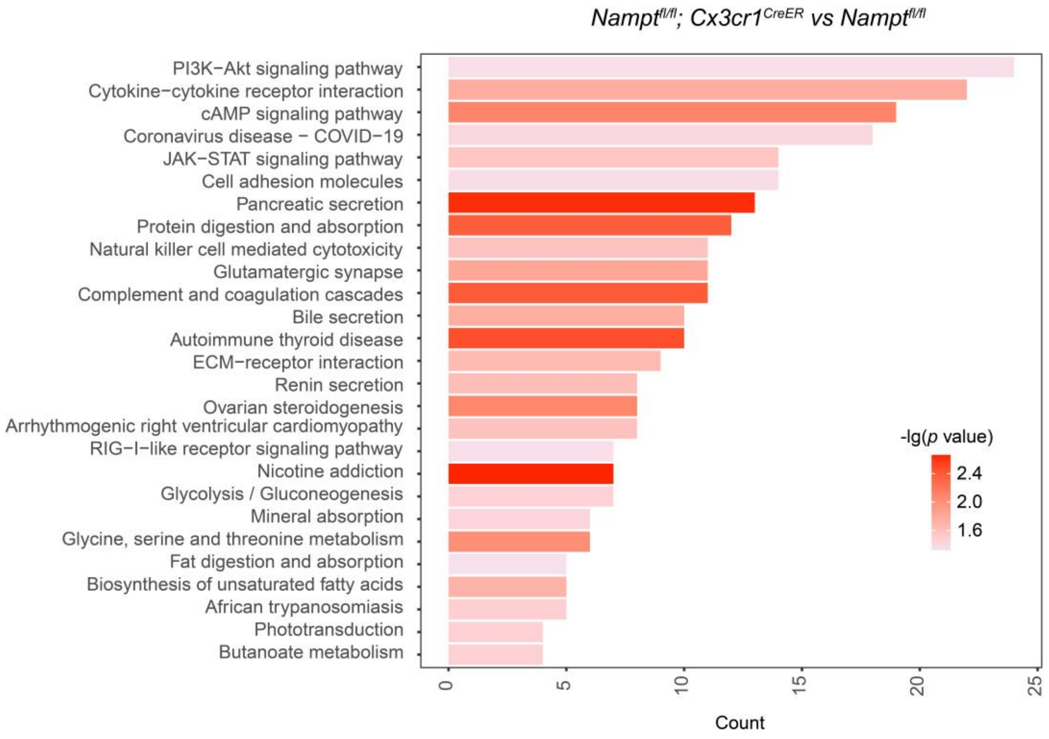

Figure S6 A KEGG enrichment analysis of differentially expressed genes between monocyte/macrophage NAMPT-specific deletion mouse (*Nampt*$^{fl/fl}$;*Cx3cr1*^{CreER}) and wild type mouse (*Nampt*$^{fl/fl}$).

The vertical axis represents the cell signaling pathway category, and the horizontal axis represents the enriched gene count. The color key represents the negative of log 10 (*p* value).

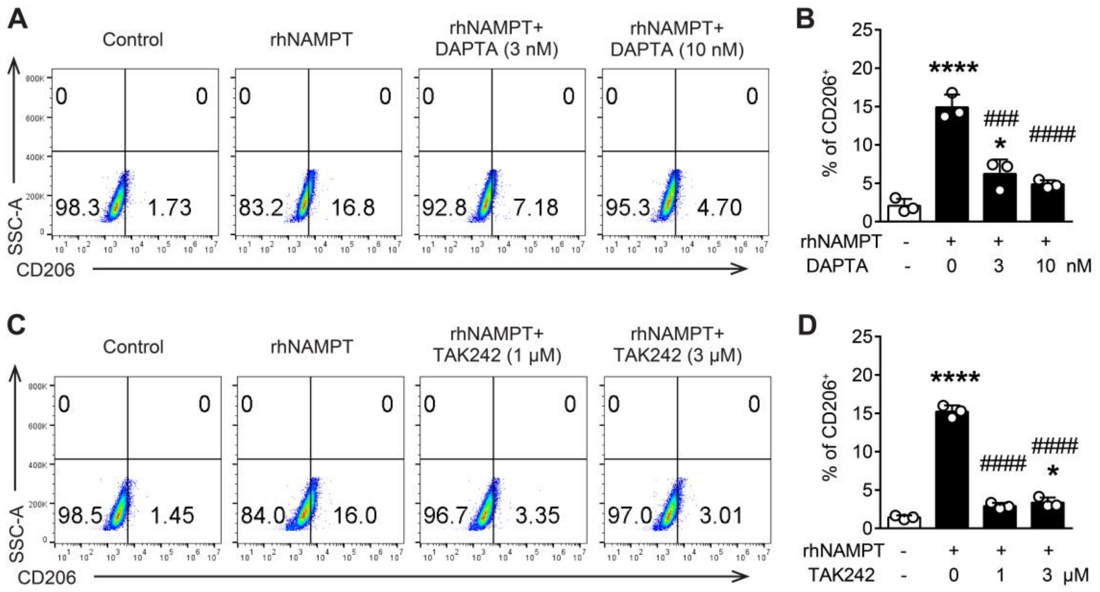

Figure S7 Extracellular NAMPT-induced M2 polarization may be mediated by CCR5 and TLR4.

RAW264.7 cells were treated with CCR5 blocker DAPTA or TLR4 blocker TAK242 for 1 h, and then rhNAMPT (300 nM) was added. The CD206$^+$ cells were detected using flow cytometry. Mean ± SD (n = 3). $^{*}P < 0.05$, $^{****}P < 0.0001$, compared with Control; $^{\#\#\#}P < 0.001$, $^{\#\#\#\#}P < 0.0001$, compared with rhNAMPT, one-way ANOVA.
